# Plasticity of 3D Hydrogels Predicts Cell Biological Behavior

**DOI:** 10.1021/acs.biomac.4c00765

**Published:** 2024-11-08

**Authors:** Andrea Malandrino, Huijun Zhang, Nico Schwarm, David Böhringer, Delf Kah, Christian Kuster, Aldo R. Boccaccini, Ben Fabry

**Affiliations:** † Biomaterials, Biomechanics and Tissue Engineering Group, Department of Materials Science and Engineering and Research Center for Biomedical Engineering, Universitat Politècnica de Catalunya, Barcelona 08019, Spain; ‡ Institute of Biomaterials, Department of Material Science and Engineering, Friedrich-Alexander University Erlangen-Nürnberg, Erlangen 91058, Germany; § Biophysics Group, Department of Physics, Friedrich-Alexander University Erlangen-Nürnberg, Erlangen 91052, Germany

## Abstract

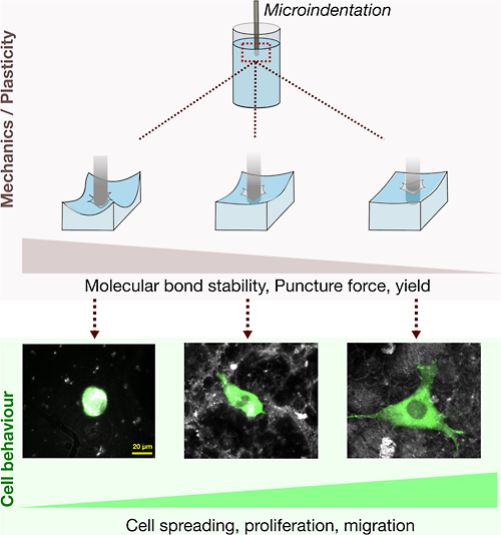

Under 3D culture conditions, cells tend to spread, migrate, and proliferate better in more viscoelastic and plastic hydrogels. Here, we present evidence that the improved cell behavior is facilitated by the lower steric hindrance of a more viscoelastic and plastic matrix with weaker intermolecular bonds. To determine intermolecular bond stability, we slowly insert semispherical tipped needles (100–700 μm diameter) into alginate dialdehyde-gelatin hydrogels and measure stiffness, yield strength, plasticity, and the force at which the surface ruptures (puncture force). To tune these material properties without affecting matrix stiffness, we precross-link the hydrogels with CaCl_2_ droplets prior to mixing in NIH/3T3 fibroblasts and final cross-linking with CaCl_2_. Precross-linking introduces microscopic weak spots in the hydrogel, increases plasticity, and decreases puncture force and yield strength. Fibroblasts spread and migrate better in precross-linked hydrogels, demonstrating that intermolecular bond stability is a critical determinant of cell behavior under 3D culture conditions.

## Introduction

Bioprinting is a method of biofabrication in which living cells are suspended in a highly viscous or pasty matrix, forming a bioink. After extrusion of the bioink through the nozzle of a 3D printer, followed by cross-linking or polymerization for mechanical fixation, the resulting biofabricate should have appropriate structural, mechanical, and adhesive properties to provide a biocompatible environment for cells. One of the key challenges is to optimize and tailor the physicochemical properties of the biofabricate to achieve desired cell behavior, such as cell proliferation and colonization of the matrix, cell differentiation, cell migration, and endothelialization. This requires a thorough understanding of the interactions between the cells and bioprinted material.

A widely used and versatile class of bioinks with tunable properties are alginate-based hydrogels.^1^ Alginate is a biocompatible natural polysaccharide. It is inexpensive, has good printability and extrusion fidelity, is largely bioinert and nonadhesive, and can be cell-friendly cross-linked with Ca^2+^ ions. Alginate can be bioprinted as-is, or it can be oxidized to alginate dialdehyde (ADA), whereby aldehyde groups along the polysaccharide backbone are used as linkers for further chemical modification.^2^ For example, amino groups of cell adhesion proteins such as collagen or laminin can be covalently attached to the aldehyde groups through a Schiff’s base reaction by simply mixing the desired protein with ADA prior to cross-linking. To further improve the biocompatibility and degradation properties of ADA, high concentrations (2–5 wt %) of gelatin (GEL) can be added to the mixture, followed by cross-linking with transglutaminase, to form an ADA-GEL copolymer.^3−5^ Gelatin further improves the adhesion properties of the bioink, is far less expensive compared to purified extracellular matrix proteins, and is stable at room temperature.

When cells such as dermal fibroblasts are seeded on top of ADA-GEL hydrogel surfaces, they readily attach, spread, and proliferate.^5^ By contrast, when the cells are mixed into the ADA-GEL matrix, we found that they spread and proliferate poorly, although they remain viable.^6^ Precross-linking the ADA-GEL bioink with low to moderate concentrations of Ca^2+^ ions prior to extrusion and post cross-linking greatly improved cell migration, spreading, and proliferation.^6^ Precross-linked ADA-GEL hydrogels were similarly stiff compared to non-precross-linked ADA-GEL hydrogels but displayed a more viscoelastic behavior.^6^ This finding was in agreement with previous reports claiming that increasing the viscous behavior (or equivalently the stress relaxation time constant) of otherwise similarly stiff viscoelastic hydrogels improved cell biocompatibility.^7,8^ Furthermore, precross-linked ADA-Gel is one of the few extrusion-printable bioinks that support rapid cell proliferation and invasion/migration comparable to nonprintable biopolymer hydrogels (e.g., Matrigel, collagen).

We discovered that with increasing degree of precross-linking, the ADA-GEL hydrogels became not only more viscoelastic but also more plastic, as demonstrated by the degree of stress relaxation after applying a 5% compressive strain for 10 min. Changes in cell proliferation, spreading, and migration were more closely correlated with hydrogel plasticity than with the viscoelastic relaxation time constant. This is consistent with other reports that have found that different patterns of plastic remodeling regulate biological outcomes such as fibroblast activation,^9^ cancer cell migration,^10^ and vascular assembly and invasion.^11^ In the present work, plasticity refers to the inelastic, irreversible matrix deformations that result from the mechanical breakage of intermolecular bonds. Since alginate-based hydrogels are not degraded by cell-secreted proteases,^12^ cells must mechanically break matrix bonds by force application in order to spread, migrate, and proliferate. Therefore, we reasoned that plasticity may be a reliable predictor of cell behavior because it reports the mechanical stability of the material’s intermolecular bonds.

Here, we provide more direct evidence that precross-linking of ADA-GEL hydrogels reduces the force at which intermolecular bonds in the hydrogel break. To demonstrate this, we puncture the surface of ADA-GEL hydrogels with blunt needles and record the force at which the hydrogel surface ruptures and the needle penetrates the 3D matrix, in addition to confirming with new precross-linking methods that increased plasticity correlates to biological behavior. We then correlate the puncture forces of different hydrogels with the spreading and migration behaviors of cells grown in these hydrogels. We conclude that puncture force experiments provide a simple and novel method to estimate the protrusive cell forces required for spreading and migration and may aid in the design of novel bioinks for improved cell behavior.

## Experimental Section (Materials and Methods)

### Hydrogels

To prepare the ADA-GEL mixture, 4.5% (w/v) ADA was dissolved in Dulbecco’s modified Eagle’s medium (DMEM) at room temperature overnight. Similarly, 9% (w/v) gelatin was dissolved in DMEM at 37 °C. The two solutions were then combined in a 1:1 ratio, resulting in a solution containing 2.25% ADA and 4.5% gelatin.

We then precross-linked the ADA-GEL bioink either by nebulizing an aqueous CaCl_2_-solution onto the surface of the bioink, which was constantly stirred with a magnetic stirrer in a beaker, or by slow addition of the CaCl_2_ precross-linking solution under vigorous stirring. For the CaCl_2_ nebulization, we used a commercially available mesh nebulizer (FEELLIFE Inhalator Air Pro VIII) with an interchangeable nozzle that delivers an aerosol through a membrane driven by a piezo element. We set the nominal nebulization rate to around 0.5 mL per minute. New nozzles were used when the performance of the nebulizer visibly decreased. To extend the lifetime of the nozzle, the nebulizer was cleaned after each experiment with a 4% acetic acid solution. For the vigorous stirring of the CaCl_2_ precross-linking solution, we used an electric stirrer at maximum speed (Arendo, 14,200 rpm motor).

For ADA-GEL control conditions, sterile water was nebulized, or slowly added under vigorous stirring, onto the ADA-GEL mixture at a volume ratio of 1:8 (nebulized solution to ADA-GEL bioink). For nebulization-based precross-linking, a 360 mM or 540 mM solution of CaCl_2_ in sterile water was nebulized at a volume ratio of 1:8 (nebulized solution to ADA-GEL bioink) until a final CaCl_2_ concentration of 40 mM (low precross-linking) or 60 mM (high precross-linking) in the bioink was reached. In a typical experiment, 1 mL of CaCl_2_ solution was nebulized into 8 mL of ADA-GEL solution for approximately 5–10 min in a 50 mL beaker. During nebulization, the beaker was kept in a water bath at 37 °C with continuous stirring at a speed of 400 rpm. As the alternative technique of slow addition of CaCl_2_ with vigorous stirring allowed us to fine-tune the concentrations, we also added two further precross-linking conditions with CaCl_2_ solutions of 180 mM and 720 mM in a volume ratio of 1:8 until final CaCl_2_ concentrations of 20 mM and 80 mM were reached.

After precross-linking but prior to cell embedding, 0.4% w/v transglutaminase (Würzteufel GmbH, with an enzymatic activity of 100 units/g) was added to 8 mL of ADA-GEL mixture (and 1 mL of precross-linking solution) to enzymatically cross-link the gelatin. The final concentration of transglutaminase corresponds to an enzymatic activity of 10 units per gram of protein (in this case, gelatin), which was previously found to represent an optimal balance between gel strength and gelation time.^13^

**Figure 1:**
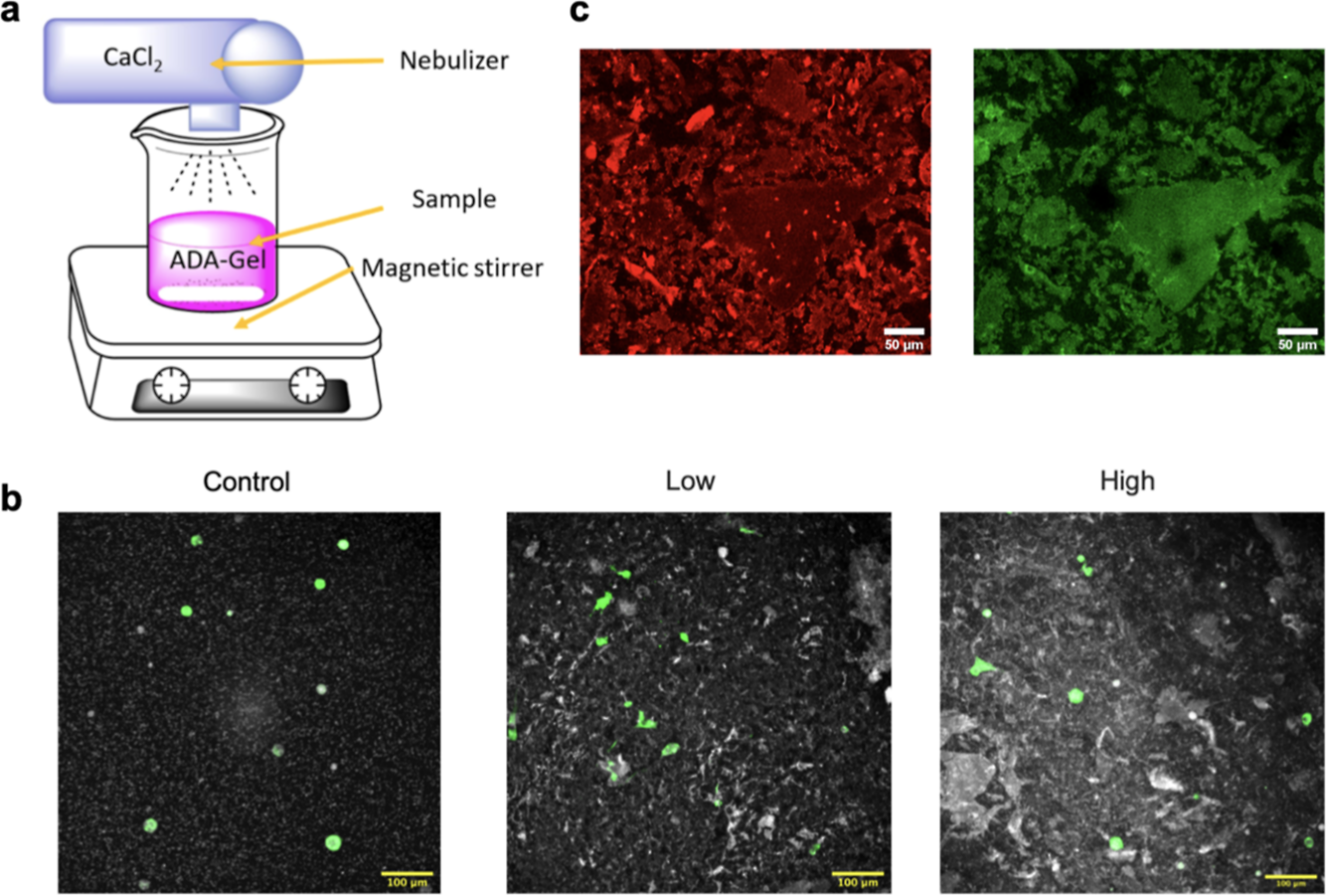
ADA-GEL precross-linking and change in confocal reflection profile. (a) Schematics of the precross-linking procedure with the use of a nebulizer under continuous stirring during the gel preparation, (b) representative confocal images (maximum projection over a 100 μm depth) of NIH/3T3-tdTomato cells embedded in ADA-GEL blends with different precross-linking degrees (low = 40 mM CaCl_2_, high = 60 mM CaCl_2_) after 3 days in culture. Images show the superposition of the cell fluorescence signal (green) and the reflection (gray), and (c) confocal images of the ADA-GEL blend with FITC-labeled Gelatin for the highly precross-linked (60 mM CaCl_2_) case. Left and right pictures show the reflection and the FITC fluorescence, respectively.

In the cell experiments, 10^5^ cells per milliliter of ADA-GEL were then added and carefully mixed using a displacement pipet. For final cross-linking, an equal volume of 100 mM CaCl_2_ in water with 5% w/v transglutaminase was pipetted carefully onto the ADA-GEL for 10 min. The hydrogel samples were then washed with HEPES and placed in standard cell incubators (37 °C, 5% CO_2_). Samples were imaged in confocal reflection and fluorescence modes using an upright confocal laser scanning microscope equipped with a 1.0 NA 20× dip-in objective (Leica SP5, Leica, Wetzlar). For the confocal reflection profiles shown in Figures 1 and S1 (Supporting Information), we relied on the principle that higher cross-linking densities cause alginate-based hydrogels to scatter more light compared to uncross-linked or weakly cross-linked alginate-based hydrogels. However, it was not possible to distinguish between reflected and backscattered light, as both were detected in confocal reflectance imaging.

### Preparation of Fluorescein Isothiocyanate-Labeled Gelatin

2g portion of gelatin was dissolved in 20 mL of 0.1 M carbonate-bicarbonate buffer (pH 9.0) at 40 °C. Then, 80 mg of fluorescein isothiocyanate (FITC) was dissolved in 300 μL of DMSO. Gelatin and FITC solution were mixed, covered with aluminum foil, and stirred at 40 °C overnight. The final mixture was purified by dialysis for 10 days in PBS to remove unreacted FITC. PBS was changed once a day. Gelatin-FITC was freeze-dried for further cell study.

### Swelling Ratio and Water Uptake Test

All precross-linked ADA-GEL hydrogels were prepared as cylindrical tablets weighing initially around 200 mg and were placed inside cell strainers, immersed in DMEM, and placed in a cell incubator (37 °C, 5% CO_2_) for up to 25 days. At specific time points *t*, the weight *w*(*t*) of the hydrogel samples was measured after unbound water was blotted with sterilized cellulose wipes. The swelling ratio was calculated according to 100[*w*(*t*) – *w*(*t* = 0)]/*w*(*t* = 0).

### Extrusion Fidelity Test

To evaluate the extrusion stability of precross-linked hydrogels, 3D structures presented in Figure S8 (Supporting Information) were printed onto a Petri dish at temperatures of 23 and 37 °C through a blunt needle with a 0.4 mm inner diameter (Braun Sterican Kanuele 21G × 10.4 × 25 mm). During printing, the printhead moved at a speed of 2.5 mm/s while depositing a volume of 0.125 μL/mm. The needle tip was positioned approximately 0.2 mm above the Petri dish.

**Figure 2:**
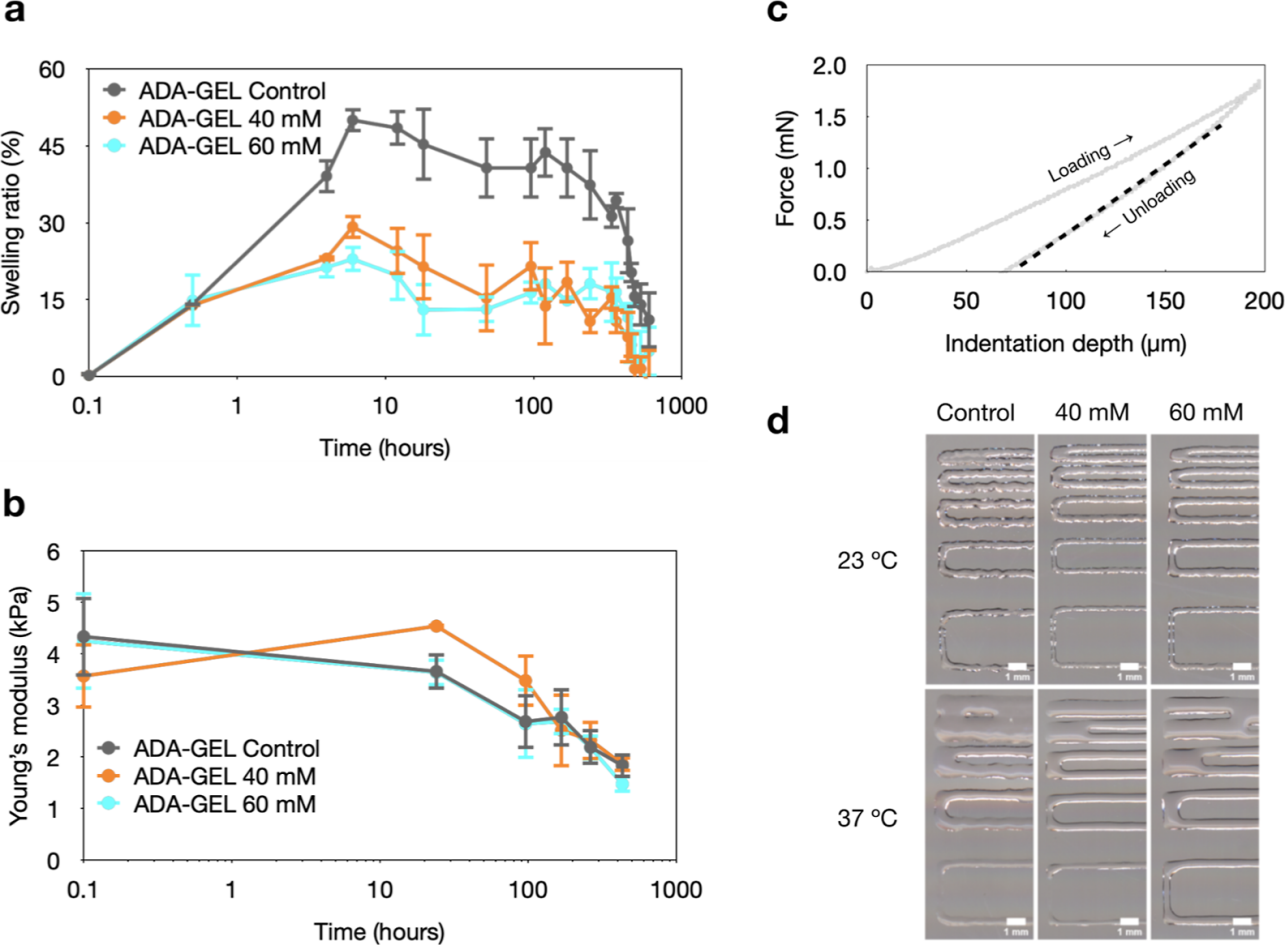
Mechanical measurements and extrusion stability of ADA-GEL with different degrees of precross-linking. (a) Swelling ratio of ADA-GEL with different precross-linking degrees immersed in DMEM at 37 °C in the cell culture incubator. Data are calculated using the hydrogels’ wet weight (*n* = 3 hydrogel samples each group, mean values ± SD), (b) Young’s modulus of ADA-GEL with different precross-linking degrees measured at room temperature over 18 days. Between the measurements, the samples are immersed in DMEM at 37 °C, (c) representative force versus indentation curve of the ADA-GEL sample to show the linear fitting performed in the unloading phase for the determination of Young’s moduli in (b). (d) Extrusion stability of ADA-GEL blends with different precross-linking degrees at 23 and 37 °C.

Printing syringes were loaded with bioink immediately after adding 0.4% w/v transglutaminase and were then stored for 30 min at 23 or 37 °C before printing. After printing, the printed patterns were cross-linked, as described above. An Epson Perfection 3200 Photo scanner with a resolution set to 1200 dpi was employed for imaging the printed structures (Figure 2d).

### Microindentation Test

For stiffness measurements using the microindentation technique, ADA-GEL hydrogels without embedded cells were cast in porous plastic rings with an inner diameter of 6 mm and a height of 8 mm or in plastic rings with an inner diameter of 10 mm and a height of 8 mm. After final cross-linking, the samples were submerged in DMEM and measured at 23 °C. For time course measurements (after 1, 4, 7, 11, and 18 days), the samples were stored in DMEM in a cell incubator (37 °C, 5% CO_2_) between measurements.

During the microindentation experiment, the hydrogel surface was indented with a cylindrical indenter (diameter = 3 mm) attached to a micromanipulator (Eppendorf InjectMan NI 2), and the indentation force as a function of indentation depth was measured with a precision laboratory scale (Sartorius Practum 64-1S). The maximum indentation depth was 200 μm, and the speed of the indentation was 5 μm/s. The force and depth of the indentation were continuously measured at a frequency of 5 Hz. The slope *S* of the force–indentation relationship during the unloading phase was then linearly fitted for indentation depths between 20 and 120 μm from the maximum depth reached (Figure 2c).

The Young’s modulus was calculated using the Hayes equation^14^ as a function of the slope of the force–indentation relationship *S*, the indenter radius *r*, Poisson ratio ν that is assumed to be 0.5 for ADA-GEL, and a geometrical correction term κ that depends on the sample height *h*, *r*, and ν:
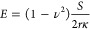
with
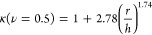


Values from three measurement positions near the sample center were averaged.

### Surface Puncture Force

Puncture force experiments were performed with acupuncture needles with diameters of 0.1 and 0.3 mm (Seirin Corporation) or 0.7 mm (wandrey GmbH). The needle tips were sanded to a hemispherical shape. ADA-GELs without embedded cells were casted into 96-well dishes (volumes of 300 μL) and cross-linked as described above. Samples were washed with DMEM and placed on a laboratory scale. The needles were mounted to a micromanipulator (Eppendorf InjectMan NI 2) and lowered toward the ADA-GEL surface at a speed of 75 μm/s until a weight of 5 μg was reached, indicating that the needle tip reached the gel surface. From this point forward, the gels were indented to a depth of 3 mm at a speed of 50 μm/s. The weight and depth of the indentation were measured continuously at a frequency of 5 Hz.

Force increased steadily for the initial 1–2 mm of indentation and then suddenly dropped when the indenter tip punctured the gel’s surface. We defined the puncture force as the maximum force reached before falling abruptly, as illustrated in Figure 5b. After exceeding the puncture force, the penetration force exhibited a fluctuating rise with increasing indentation depth, with more pronounced fluctuations for precross-linked gels. We observed an approximately linear relationship between needle diameter and puncture force (Figure 5c), consistent with a previous study.^15^

### Elasto-Viscoplastic Tests and Parameter Evaluation

To evaluate the elasto–viscoplastic parameters, we performed force–indentation experiments using an acupuncture needle with a diameter of 0.3 mm (Seirin Corporation). The needle tip was sanded to a hemispherical shape. The indentation was controlled by mounting the needle on a spindle, driven by a stepper motor, and the force was measured using a 100 g range miniature straight bar load cell.

ADA-GELs were poured into 3 individual wells (300 μL per well), broken off from a 96-well plate, and cross-linked as described above. Samples with embedded cells were placed in an incubator for 3 days and replenished with DMEM before testing. Samples were measured at room temperature. We developed a protocol in which the puncture needle is repeatedly inserted into the hydrogel and then withdrawn at progressively larger indentation depths to measure Young’s modulus, viscoelastic behavior, yield strength, and plastic deformation within the same experiment (Figure 6). The speed for inserting and withdrawing the needle was 50 μm/s for all cycles. To measure the Young’s modulus, we selected three cycles with small indentation depth (500, 600, 700 μm, Figure 6a) and performed a fit to the indentation curve (Figure 6b,c) using the Hertz theory in which the force *F* (converted from the measured weight) corresponds to
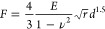
where *E* is the Young’s modulus of the hydrogel (which is assumed to be much smaller than the Young’s modulus of the indenter), ν is the Poisson ratio of the hydrogel (which is assumed to be 0.5), *r* is the indenter radius, and *d* is the indentation depth.

To characterize viscoelastic relaxation, we performed a stress relaxation experiment after indenting the needle to a depth of 800 μm and holding the position for 5 min. The force relaxation versus time (Figure 6b,c) was fitted using a stretched exponential function
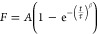
where *A*, τ, and β are the amplitude, relaxation time constant, and stretching exponent, respectively. Finally, the residual deformation (or yielding distance) after withdrawing the needle from the sample at a threshold force corresponding to 50 mg of weight was plotted against the maximum indentation reached during the same cycle (Figure 6b,c).

### Cell Behavior

For cell morphology and migration, we used NIH-3T3 fibroblasts stably expressing the red-fluorescent tandem dimer (td) tomato protein carrying a CAAX sequence that recruits the protein to the cell membrane upon farnesylation. NIH-3T3 tdTomato-farnesyl cells were generated by lentiviral transduction of the tdTomato-farnesyl-5 reporter construct as described.^16^ NIH-3T3 tdTomato-farnesyl cells (hereafter referred to as NIH-3T3 tdTomato cells) were grown in 75 cm^2^ flasks in phenol red containing DMEM supplemented with 4.5 g L^–1^ glucose, 10% (v/v) bovine calf serum, 1 U L^–1^ penicillin–streptomycin, 4 mM l-glutamine, and 1 mM sodium pyruvate.

**Figure 3:**
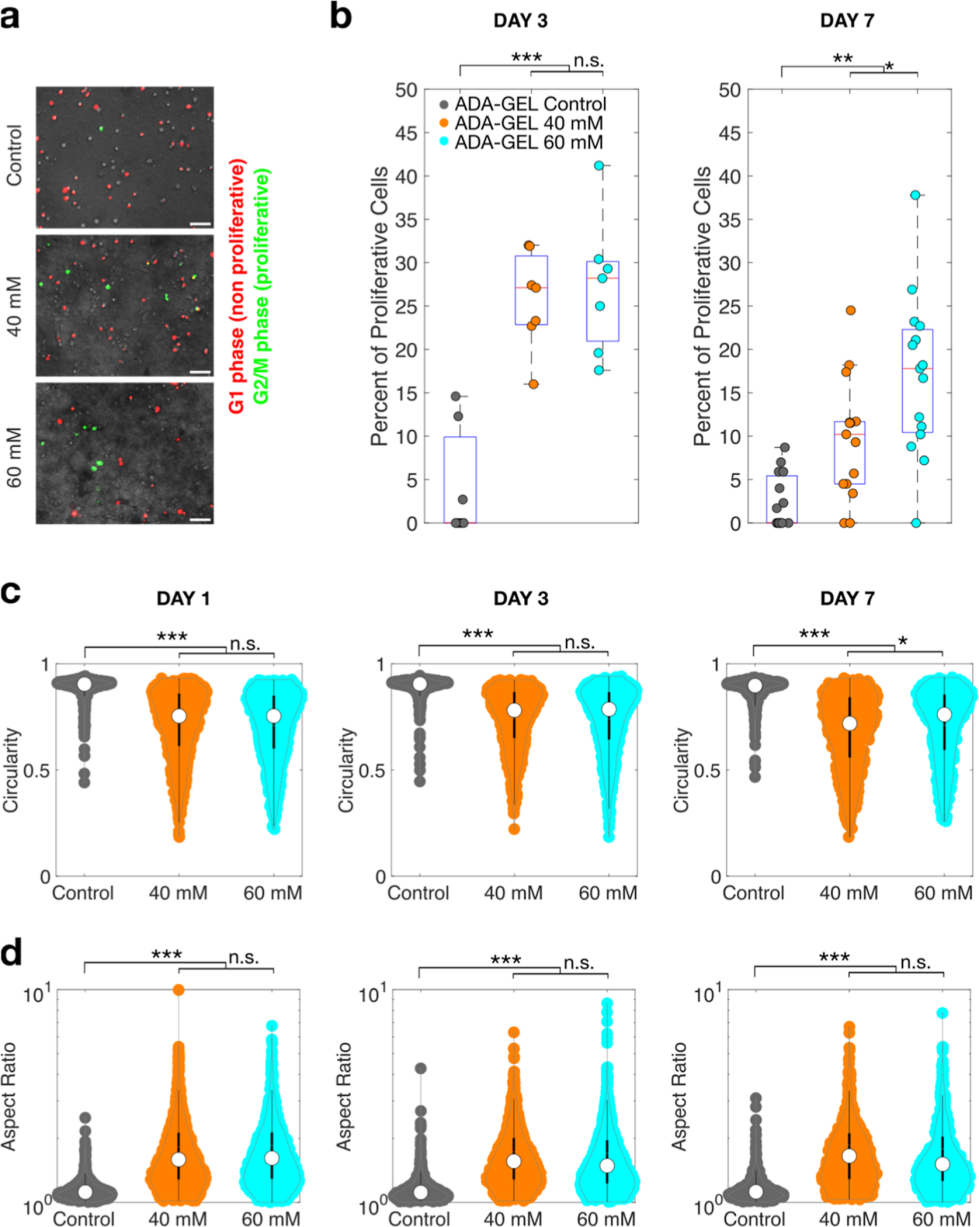
Cell morphological and proliferative behavior for ADA-GEL with different degrees of precross-linking. (a) Representative images of NIH/3T3-FUCCI cells nuclei detected in G1 (red) or G2/M (green) phase of the cell cycle, used for quantification of proliferative behavior. The nuclei signal is binarized and superimposed to maximum intensity projected brightfield images for control and low and high precross-linked ADA-GEL cases, (b) bar plots of NIH/3T3-FUCCI cell proliferation quantification for control and low and high precross-linked ADA-GEL cases. The graphs show the percent of proliferative cells (cell nuclei in G2/M phase) with respect to the total of cell nuclei (both in G1 and G2/M phases) detected by a fluorescent microscope, (c) Violin plot of cell circularity of the NIH/3T3 cells for control and low and high precross-linked ADA-GEL cases, with mean (white dot) and bandwidth (black line) between the 10% and 40% data range, on days 1, 3, and 7, (d) Violin plot of aspect ratio (log scale) of the NIH/3T3 cells for control and low and high precross-linked ADA-GEL cases, with mean (white dot) and bandwidth (black line) between the 10% and 40% data range, on days 1, 3, and 7. Statistical differences between different ADA-GEL were tested using ANOVA (* = *p* < 0.05, ** = *p* < 0.005, *** = *p* < 0.0005, n.s = *p* > 0.05). Scale bar = 100 μm.

To measure cell morphology, we obtained fluorescent image stacks of NIH-3T3 tdTomato cells embedded in ADA-GEL. Image stacks were then maximum-intensity projected, utilizing a bandpass filter to eliminate background noise, and binarized using ImageJ software (https://imagej.nih.gov).^17^ We then used the “analyze particles” plugin of the ImageJ software to measure the circularity and the aspect ratio of at least 30 individual cells in each sample from at least two and up to seven fields of view depending on the number of cells in the image (Figure 3c,d). Three samples were measured for each condition and time point.

Cell migration was measured using bright-field time-lapse imaging at day 0, day 3, and day 6 after seeding. Samples are transferred to a motorized microscope (Applied Scientific Instrumentation, USA), equipped with a 4× magnification objective (Olympus, Japan) and a CCD camera (Lumenera Infinity 3-6UR, Canada), placed in a tissue culture incubator (37 °C, 5% CO_2_). Two independent experiments were conducted, with three/four wells per condition (control, 40 mM, and 60 mM precross-linked), and at least three positions per well were measured. An average of ∼800 cells were tracked at each position. Unstable or excessively drifting positions were excluded. In total, there were 16, 24, and 24 positions for control, 40 mM, and 60 mM, respectively, on day 3 and 21, 24, and 17 on day 6. At each position, we recorded minimum intensity-projected images from image stacks (volume of each stack, 2818 × 2244 × 70 μm^3^, voxel size, 1.02 × 1.02 × 10 μm^3^) acquired every 15 min over a period of 23 h. Cell migration was analyzed in a similar manner to that described in refs (6,18). In brief, cell positions in each minimum intensity-projected image are detected using Li-thresholding,^19^ and cell trajectories between subsequent images are stitched based on Euclidean distance and migration direction criteria. To ensure reliability, cell trajectories shorter than 4 h were disregarded for further analysis. Cells were classified as motile if they migrated a minimum of 20 μm from their starting position during the entire observation period. Cell speed was calculated between consecutive frames (15 min apart) and averaged over the measurement period for each cell. We also calculated for each cell the total travel distance over 1 h trajectory segments, as well as the turning angles between consecutive 1 h trajectory segments. Persistence values were then calculated as the cosine of the turning angles and were averaged over the measurement period for each cell. Finally, we computed the average speed, persistence, and travel distances over all cells within a field of view.

To measure cell proliferation, we used NIH-3T3 cells transfected with the two-color fluorescent cell cycle indicator Fucci.^20^ Fucci-transfected cells were cultured under conditions identical to those listed above for NIH-3T3 and tdTomato cells. Fucci-transfected cells show high red and low green fluorescence when they are in the G1 phase of their cell cycle and low red but increasing levels of green fluorescence as they progress through S, G2, and early M phases.^17^ The image analysis protocol for extracting the percentage of proliferating cells included maximum intensity projections of epifluorescence-acquired image stacks, contrast enhancement, band-pass filtering, background removal, and binarization. Both green and red nuclei were then counted, and the percentage of proliferating cells (Figure 3a,b) was computed as the ratio of green nuclei (S, G2, and M phases) to the total number of red and green nuclei counted. A minimum of 30 cells from two to seven fields of view were analyzed per sample, and three samples were measured for each condition and time point.

## Results and Discussion

### Results

: Precross-linking ADA-GEL with Ca^2+^ ions results in similar mechanical properties but changes their microstructure.

We modified a previously established method for precross-linking ADA-GEL with Ca^2+^ ions to achieve improved optical clarity: instead of mixing ADA-GEL with CaCO_3_ particles,^6^ we directed an ultrasound-nebulized stream of CaCl_2_ solution droplets onto an ADA-GEL mixture. In order to homogenize the dispersion of droplets, the mixture was continuously stirred during the nebulization process (Figure 1a). The nebulization time was adjusted to obtain a lower (40 mM) or higher (60 mM) total concentration of CaCl_2_ in the mixture. A control condition is obtained by nebulizing water into the mixture. As a final step, the ADA-GEL mixtures were enzymatically and ionically cross-linked with transglutaminase (4 mg/mL) and 100 mM CaCl_2_ in distilled water (see Experimental Section (Materials and Methods)). The precross-linked ADA-GEL showed a heterogeneous structure with differently reflective regions and spots when observed through a confocal reflection microscope (Figure 1b,c). The size of these reflective spots increased with the degree of precross-linking (Figure 1b). From the observation that alginate hydrogels scatter more light with an increasing degree of cross-linking, we conclude that precross-linking with CaCl_2_ droplets introduces a separation of the hydrogel into regions with a higher or lower degree of ADA-cross-linking.

We prepared ADA-GEL hydrogels with FITC-stained gelatin to measure the homogeneity of the gelatin phase. We then compared the confocal reflectance and fluorescence signals of the same region (Figure 1c). We found that few hydrogel regions have both high reflectance and fluorescence, but most other regions showed no clear colocalization of the reflectance and fluorescence signals. In particular, smaller spots were mostly seen in the reflectance channel but not in the fluorescence channel. From this, we conclude that precross-linking introduces regionally uncorrelated inhomogeneities in both the degree of cross-linking and the concentration of gelatin.

Next, we investigated the macroscopic mechanical behavior of ADA-GEL that was not precross-linked, pre-crosslinked with 40 mM CaCl_2_ and 60 mM CaCl_2_. First, the swelling and degradation properties of the ADA-GEL hydrogels were analyzed by measuring the specific weight *w* of the three hydrogel formulations over 25 days. As shown in Figure 2a, the swelling ratio of ADA-GEL hydrogels (the ratio *w*(*t*)*/w*(*t* = 0)) rapidly increased during the first 6 h after immersion in DMEM and then decreased slowly over the following days but decreased more rapidly after day 18. The peak swelling ratio measured after 6 h was highest—about 50%—for the control (nonprecross-linked) ADA-GEL and lower—about 20–30%—for the precross-linked gel. The swelling ratio followed similar trends in all three hydrogel formulations.

Next, we measured the mechanical properties of the hydrogels using microindentation with a 3 mm diameter indenter. These measurements, carried out over a period of 18 days, showed that the stiffness (Young’s modulus) was similar for all conditions (Figure 2b). Young’s moduli were obtained through linear fitting of the force–indentation curves in the unloading phase (Figure 2c and Experimental Section (Materials and Methods)). Stiffness was highest immediately after final cross-linking (at day 0, 3.6 kPa for the control samples and 3.1 kPa for both precross-linked samples) and decreased similarly for all conditions over time when immersed in DMEM, regardless of their degree of precross-linking.

Given the importance of alginate-based hydrogels as bioinks in tissue engineering, we also investigated the extent to which extrusion stability is dependent on the degree of precross-linking. Figure 2d shows that the extrusion stability was better at 23 °C compared to that at 37 °C. In addition, the precross-linked conditions showed slightly improved extrusion stability at 23 and 37 °C compared to the control group. With a 200 μm printing capillary, the print resolution at 23 °C was 0.5 mm for the 40 and 60 mM conditions and 0.6 mm for the control condition, while at 37 °C, the print precision was 0.7 mm for the 40 and 60 mM conditions and 0.8 mm for the control group.

### Precross-Linking ADA-GEL Improves Cellular Behavior

We evaluated the biological properties of ADA-GEL precross-linking by performing morphological, proliferation, and migration assays of NIH-3T3 cells 3D-embedded in the gel. We used a FUCCI-based NIH-3T3 cell line as a proliferation reporter (Figure 3a). In this cell line, two proteins that are reciprocally active during the cell cycle are labeled with different fluorescent proteins so that the fluorophore visible at the nucleus indicates the phase of the cell cycle. Figure 3b shows that proliferation changed as a function of both time and the degree of precross-linking. The percentage of proliferative cells was considerably higher in the 40 and 60 mM precross-linked ADA-GEL hydrogels compared to that in control conditions. From day 3 to day 7, the percentage of proliferative cells decreased under all conditions but remained highest in 60 mM precross-linked ADA-GEL hydrogels.

Next, we evaluated the morphology of NIH-3T3 tdTomato cells embedded in the hydrogels as a function of both precross-linking and time in culture (Figures 3c,d and S1, Supporting Information). We measured circularity and aspect ratio (major axis divided by the minor axis) as two inversely related morphological parameters. Cells spreaded similarly in the two precross-linked ADA-GEL conditions as early as 1 day after embedding, whereas the cells maintained a round morphology in the nonpre-cross-linked control hydrogels. After day 1, cell morphology remained stable over time up to 7 days after embedding.

**Figure 4:**
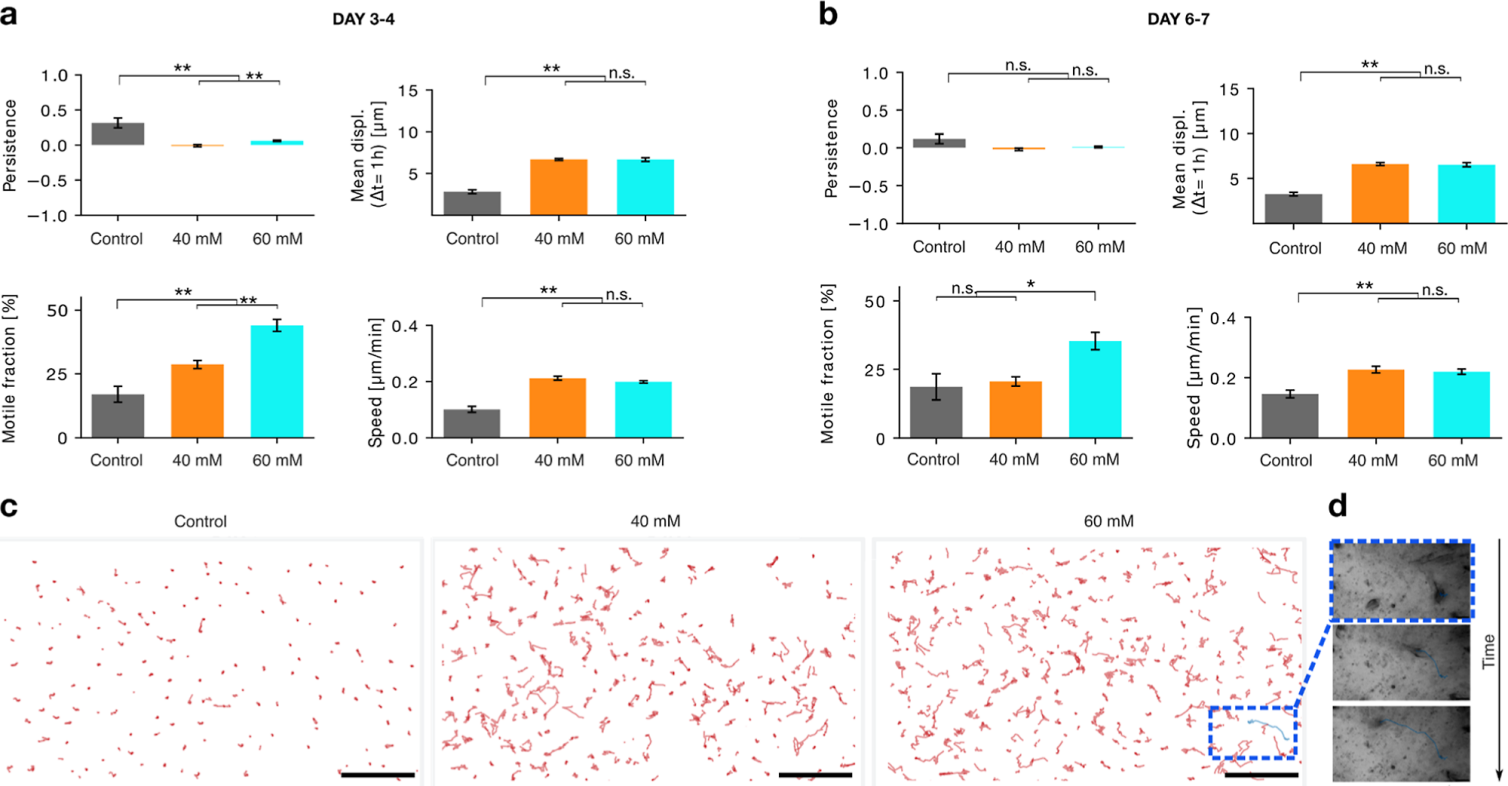
Cell migratory behavior for ADA-GEL with different degrees of precross-linking. (a) 24 h migration (mean and standard error over individual field of views, with an average of ∼800 cells tracked) of NIH/3T3 cells recorded starting at day 3 in terms of persistence, mean displacement, motile fraction, and speed for the ADA-GEL control, ADA-GEL precross-linked with 40 mM CaCl_2_ , and ADA-GEL precross-linked with 60 mM CaCl_2_ . (b) 24 h migration of NIH/3T3 cells recorded starting at day 6, (c) representative trajectories of all cells tracked over 12 h within a field of view in the ADA-GEL control, ADA-GEL precross-linked with 40 mM CaCl_2_ , and ADA-GEL precross-linked with 60 mM CaCl_2_ . The blue colored trajectory highlighted by the dotted box corresponds to the trajectory in (d). (d) Representative minimum intensity-projection brightfield images and trajectories of an individual NIH/3T3 cell migrating through the ADA-GEL precross-linked with 60 mM CaCl_2_. Statistical differences between different ADA-GEL were tested using ANOVA (* = *p* < 0.05, ** = *p* < 0.005, *** = *p* < 0.0005, n.s = *p* > 0.05). Scale bar = 200 μm.

We then measured the ability of NIH-3T3 tdTomato cells to migrate within the ADA-GEL hydrogels depending on the degree of precross-linking (Figure 4). Consistent with the increased proliferation and spreading in precross-linked ADA-GEL mixtures, the cell motile fraction and migration speed were higher in the precross-linked ADA-GEL compared to that in control, both at 3 and 6 days in culture. These differences were already manifested after 24 h of culture (Figure S2, Supporting Information). For all metrics except motile fraction, the two precross-linked conditions (with 40 and 60 mM CaCl_2_) were not significantly different from each other but showed clearly significantly different values compared to control. Motile fractions increased significantly from control to the highest degree of precross-linking at day 3, but not at day 7, when the intermediate precross-linked condition was similar to control. Cell viability over 1 week of culture was similarly high for both control precross-linked samples (Figure S3, Supporting Information).

### Precross-Linked ADA-GEL Have Lower Puncture Force and Yield Strength and Higher Plasticity

**Figure 5:**
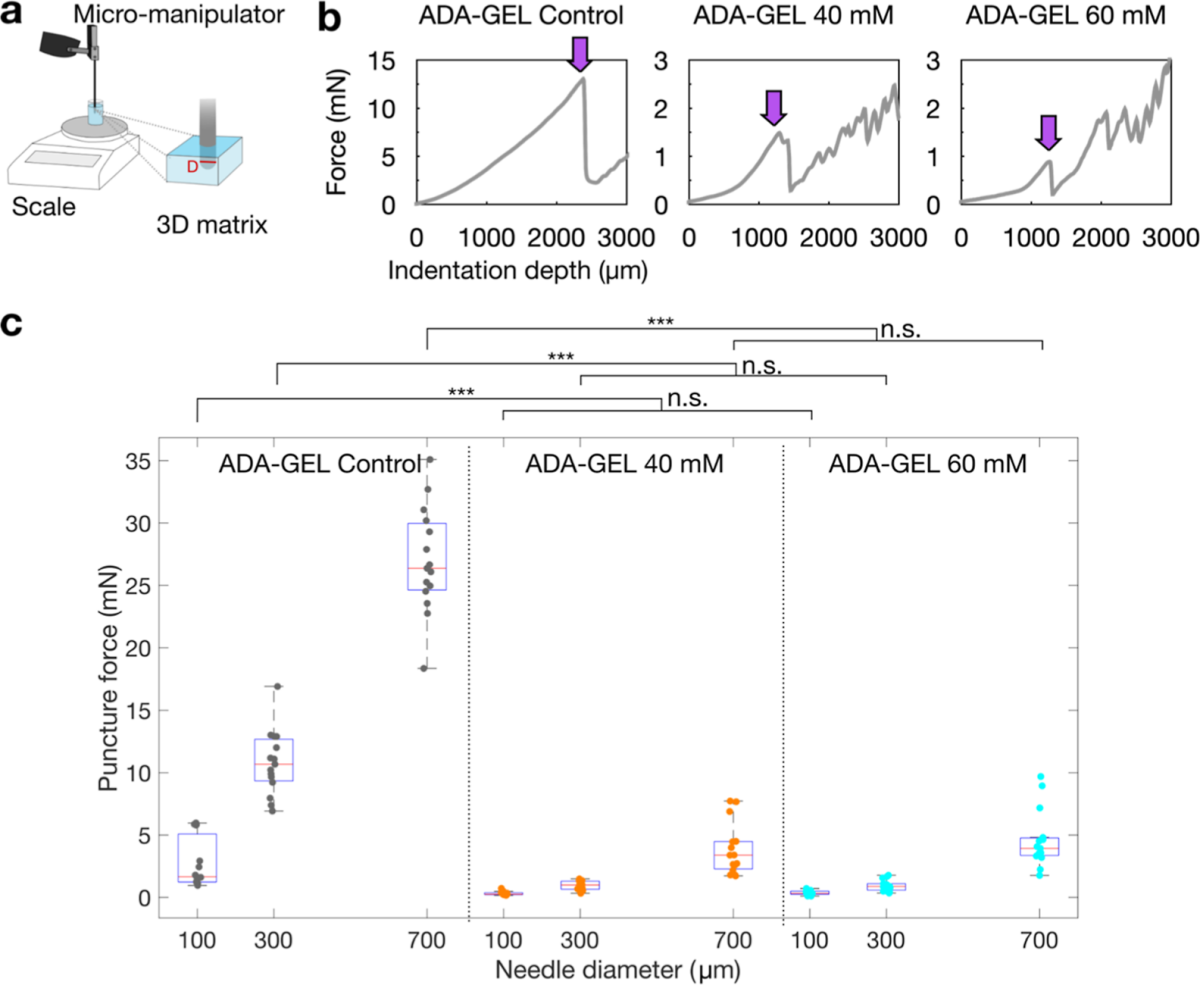
Cell–material interactions and puncture experiments to link to increased migration/spreading. (a) Experimental setup: a cylindrical steel needle with a hemispherical tip with diameter *D* was driven with a defined velocity into a ADA-GEL hydrogel. The indentation force was monitored with a standard laboratory precision scale, (b) representative force versus indentation curves measured with a 300 μm diameter hemispherical needle for ADA-GEL control, ADA-GEL precross-linked with 40 mM CaCl_2_, and ADA-GEL precross-linked with 60 mM CaCl_2_. Arrows indicate the point when the surface is punctured. (c) Surface puncture forces from *n* = 15 samples per condition and needle plotted as a function of the hemispherical tip diameter for the three conditions as boxplots indicating the median, the 25th and 75th percentiles (box), and the most extreme data points not considered outliers (whiskers). Statistical differences between different ADA-GEL were tested using ANOVA (* = *p* < 0.05, ** = *p* < 0.005, *** = *p* < 0.0005, n.s = *p* > 0.05).

To test whether the enhanced cellular behavior in precross-linked ADA-GEL was due to a lower bond strength of the matrix, we penetrated the material with blunt (semispherical tipped) needles with diameters of 100, 300, and 700 μm and recorded the corresponding force–depth profiles (Figure 5a,b). For all diameters, we found a characteristic force–depth profile (Figure 5b) where the force first slowly increased and then suddenly dropped. Concurrent microscopic images confirmed that the slow force increase was associated with a downward bending of the hydrogel surface and the sudden force decrease with a penetration of the needle through the hydrogel surface into the hydrogel whereby the surface recoiled upward (Supporting Information Videos 1–3). Following the established nomenclature, we refer to the force value immediately before the sudden drop as the “puncture force”, which is the force required to penetrate the gel surface. The subsequent force–depth profile after surface puncture was more irregular for precross-linked ADA-GEL hydrogels (Figure 5b).

Importantly, the puncture force for precross-linked ADA-GEL hydrogels was 1 order of magnitude lower than for nonprecross-linked ADA-GEL at all diameters (Figure 5c). There was no statistically significant difference between the puncture forces of the low (40 mM) and high (60 mM) precross-linked conditions.

**Figure 6:**
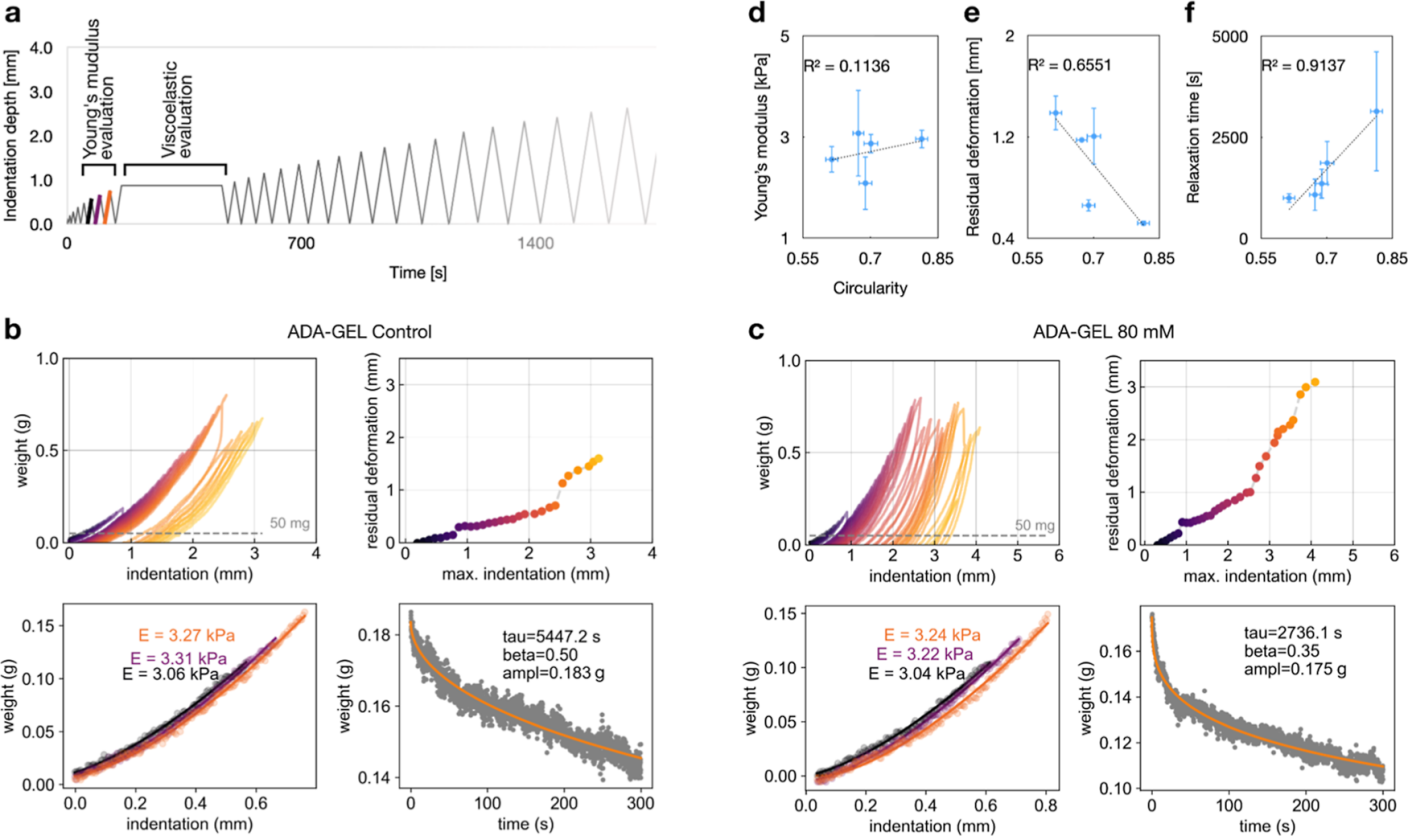
Elasto-viscoplastic profiles of precross-linked ADA-GEL hydrogels and their relation to cell spreading after 3 days in culture. (a) Experimental indentation setup: a cylindrical steel needle with a hemispherical tip of 300 μm diameter was driven into an ADA-GEL hydrogel with a defined velocity profile. The indentation force was monitored using a standard laboratory precision balance. The history of the velocity profile was designed to repeatedly insert and withdraw the tip into the hydrogel at progressively larger indentation depths to determine the plastic deformation and also to obtain the Hertz-calculated Young’s modulus (in three cycles with small indentation depth, i.e., 500, 600, 700 μm, shown) and the viscoelastic relaxation fit by keeping the indentation constant for 300 s, (b) representative profiles for the ADA-GEL control case, showing the weight vs indentation curves as the indenter was inserted and withdrawn according to the indentation history shown in (a) (top left); the residual deformation at a threshold force (corresponding to a minimum 50 mg weight threshold) versus the maximum indentation for the specific cycles is colored as in the previous weight vs indentation curves (top right); the Young’s modulus calculated by Hertz for cycles 5, 6, and 7 (bottom left), and the viscoelastic fit using a stretched exponential law (bottom right). (c) Representative profiles for the ADA-GEL precross-linked with 80 mM CaCl_2_ [see description of the plots in (b)]. (d–f) Correlations of elasto-viscoplastic parameters (Young’s modulus, residual deformation at 2 mm maximum indentation depth, and relaxation time constant of the viscoelastic fit, respectively) with cell spreading (circularity). Each point represents the mean value for a condition (control, 20, 40, 60, and 80 mM precross-linking strength) in which both cell spreading and elasto-viscoplastic parameters were measured. The bars indicate the standard error of the mean for *n* = 3 replicates of the same measurement.

To further validate our hypothesis that the improved cell behavior at higher degrees of cross-linking is due to a lower yield strength and higher plasticity of the hydrogels, we additionally used a different precross-linking method, which allowed us to add four finely tuned degrees of precross-linking to the control ADA-GEL, i.e., 20, 40, 60, and 80 mM CaCl_2_. The hydrogels were punctured using a 300 μm-diameter, hemispherical-tipped puncture needle, which was repeatedly inserted into the material and then withdrawn at progressively greater depths of penetration, typically 3 to 4 mm (Figure 6a–c). This protocol allowed us to measure Young’s modulus (for small indentation depths), viscoelastic behavior, yield strength, and plastic deformation within the same experiment after 3 days in culture with embedded cells. We first confirmed that the Young’s modulus, calculated using the Hertz theory, was unaffected by the degree of precross-linking. Conversely, viscoelastic relaxation, quantified by three parameters from a stretched exponential fit of stress relaxation over 300 s, was dependent on precross-linking, in particular the relaxation time constant. When we quantified plastic yield as residual deformation versus maximum indentation, we found an increase with the degree of precross-linking (Figures 6b,c and S4–S6, Supporting Information). We further quantified the spreading of NIH-3T3 tdTomato cells embedded in the same hydrogels after 3 days in culture using the circularity metric and confirmed an increase in spreading with the degree of precross-linking (Figure S7, Supporting Information). Taken together, we found no correlation between the elastic response (Young’s modulus) and the biological behavior (spreading) (Figure 6d) and stronger correlations with the viscoplastic response (Figure 6e,f).

### Discussion

: In this study, we investigated the relationship between hydrogel penetration force and the ability of embedded fibroblasts to spread, migrate, and proliferate. We compared alginate-based hydrogels with varying degrees of polymer chain precross-linking. Precross-linking was achieved by either nebulizing water droplets with different concentrations of Ca^2+^ ions onto the surface of a constantly stirred alginate solution or by slow addition of the CaCl_2_ precross-linking solution under vigorous stirring prior to mixing-in the cells and final cross-linking with a high (100 mM) concentration of Ca^2+^ ions. Precross-linking did not markedly change the Young’s modulus of the hydrogel after final cross-linking, and the hydrogels were stable over a 10 day period without significant degradation.

A previous report showed that precross-linking enhances the proliferation, spreading, and migration of embedded cells, and that the enhanced cell behavior correlates more strongly with the plasticity of the hydrogels and not so much with the viscoelastic relaxation time.^6^ The authors of that study speculated that the higher plasticity of the precross-linked hydrogels was attributable to a lower intermolecular bond strength of the cross-links, and that a lower bond strength allowed the cells to overcome the steric hindrance of the hydrogels more easily by employing cell-generated protrusive and traction forces. In the present study, we set out to test this idea by measuring the forces required to penetrate a blunt needle through the hydrogel surface as a more direct measure of the bond strength. To further investigate how intermolecular bonds are affected by the loading rate, we tested nonprecross-linked samples at indentation speeds of 50 and 500 μm/s and observed a slight decrease in puncture force at the higher loading rate (Figure S9, Supporting Information). This result suggests that viscoelastic or viscoplastic deformations stabilize the material at lower loading rates, allowing for a time-dependent reorganization of molecular chains that counteracts any thermally induced bond rupture dynamics, which typically decreases the rupture force at lower loading rates.

Precross-linking resulted in a heterogeneous hydrogel structure. Confocal reflection imaging in precross-linked ADA-GEL hydrogels revealed irregular spots and patches of several micrometers in diameter with increased light scattering and thus higher reflection intensities. With increased precross-linking, the number, size, and reflection signal intensity of these structures increased. Since ADA-GEL hydrogels scatter visible light when cross-linked, we speculate that these structures represent regions within the hydrogel with locally higher degree of cross-linking, and that these regions likely form when nebulized calcium chloride solution droplets come in contact with the bioink during the precross-linking step. As a result, the hydrogel is separated into phases of stronger and weaker cross-linking, which can create mechanical weak spots between the phase boundaries, allowing cells to spread and migrate. Alternatively, it is also conceivable that cells spread and migrate along or within regions with a lower degree of cross-linking;^7,21^ however, we did not observe a higher occupancy of cells in regions with lower reflection signal intensities and hence lower degree of cross-linking.

Spreading and migration require the cell to overcome the steric hindrance of the extracellular matrix. In amorphous, nonporous (relative to the size of cells and cell protrusions) materials, spreading and migration inevitably require the cells to break matrix bonds through the generation of mechanical forces.^22,23^ The forces needed to break molecular bonds in a hydrogel matrix can be estimated from the surface puncture force.^15,24^ Hence, we hypothesize that cell spreading and migration increase in hydrogels with a lower puncture force.

The study’s primary result confirms this hypothesis: cells displayed enhanced elongation, migration, and proliferation within precross-linked hydrogels, which had a much lower puncture force compared to control hydrogels (Figures 5 and S10, Supporting Information). In addition, we developed a new precross-linking protocol involving vigorous stirring, which allowed us to more homogeneously distribute the precross-linking throughout the hydrogel. In these homogeneously precross-linked hydrogels, we confirmed that the improved cell behavior at higher degrees of precross-linking was due to the lower yield strength and higher plasticity of the hydrogels (Figures 6 and S4–S6, Supporting Information). This also holds true for the highest precross-linking concentration of 80 mM CaCl_2_, where the viscoelastic and plastic material properties of the hydrogels reversed toward those seen in nonpre-cross-linked hydrogels (Figure 6d), as did the cell spreading behavior (Figure S4, Supporting Information).

Our data are in support of a previous study where we showed that cell behavior in differently cross-linked hydrogels correlates not so much with the viscoelastic stress relaxation time constant^25^ but instead with hydrogel plasticity, as measured by the nonrecoverable stress amplitude after applying a compressive strain of 5%.^6^

For an amorphous, nonporous (at the micrometer-scale) bioink such as ADA-GEL, our data show that the puncture force of a smooth surface is considerably higher than the penetration force after the surface has ruptured, and cracks have likely formed within the material. We propose that a cell embedded in a biomaterial ink must first generate high local forces to puncture the surface at the cell–material interface before the cell can elongate and migrate with lower forces. This would explain why we see not only a decrease in cell elongation and migration speed in hydrogels with higher puncture force but also a considerably lower fraction of motile cells, albeit randomly moving (as demonstrated by the persistence metric). Accordingly, the nonmotile cell fraction represents, at least to some extent, those cells that were unable to generate sufficient forces to rupture the surface at the cell–material interface.

## Conclusions

Our study introduces a novel metric, the surface puncture force, to estimate the steric hindrance encountered by cells embedded in a hydrogel. We showed that precross-linked ADA-GEL hydrogels exhibited significantly lower puncture forces, lower yield strength, and higher plasticity compared to control ADA-GEL hydrogels while at the same time exhibiting improved cell proliferation, a higher degree of spreading, and a higher proportion and speed of migrating cells. This finding suggests that plasticity, and in particular surface puncture force, which is a readily measurable parameter, predicts cell behaviors that are predominantly influenced by the steric hindrance of a 3D matrix. Our finding may help accelerate the development of novel bioinks with improved cell biocompatibility.

## Supporting Information Available

Representative single cells’ confocal images of NIH/3T3-tdTomato cells embedded in ADA-GEL blends after 3 days in culture; 24 h migration of NIH/3T3 cells in both control and highly precross-linked ADA-GEL; live/dead assays of cells embedded in ADA-GEL blends; cell spreading (all conditions) and elasto-viscoplastic profiles (precross-linked ADA-GEL hydrogel with 20, 40, and 60 mM of CaCl_2_); printing path for assessing the extrusion stability of hydrogels; puncture force of control ADA-GEL hydrogel at two different displacement rates; correlations between puncture force and biological results; and supplemental videos of puncture force experiments (ZIP)
