# Supplementary material for: Plasticity of 3D Hydrogels Predicts Cell Biological Behavior
Source: Biomacromolecules. 2024 Nov 8;25(12):7608–18. doi: 10.1021/acs.biomac.4c00765 (PMC11632650; doi:10.1021/acs.biomac.4c00765)
Supplement: Supplementary file 1 — bm4c00765_si_001.zip [file bm4c00765_si_001.zip › suppl-files/Supporting information Figures.pdf]

Supporting Information:

Plasticity of 3-D hydrogels predicts cell biological behavior

Andrea Malandrino[1*], Huijun Zhang[2], Nico Schwarm[3], David Böhringer[3], Delf Kah[3], Christian Kuster[3], Aldo R. Boccaccini[2], Ben Fabry[3*]

[1]Biomaterials, Biomechanics and Tissue Engineering Group, Department of Materials Science and Engineering and Research Center for Biomedical Engineering, Universitat Politècnica de Catalunya, Barcelona, 08019, Spain.

[2]Institute of Biomaterials, Department of Material Science and Engineering, ,Friedrich-Alexander University Erlangen-Nürnberg, Erlangen, 91058, Germany

[3]Biophysics Group, Department of Physics, Friedrich-Alexander University Erlangen-Nürnberg, Erlangen, 91052, Germany.

**Corresponding author: andrea.malandrino1@upc.edu, ben.fabry@fau.de*

Figure S1

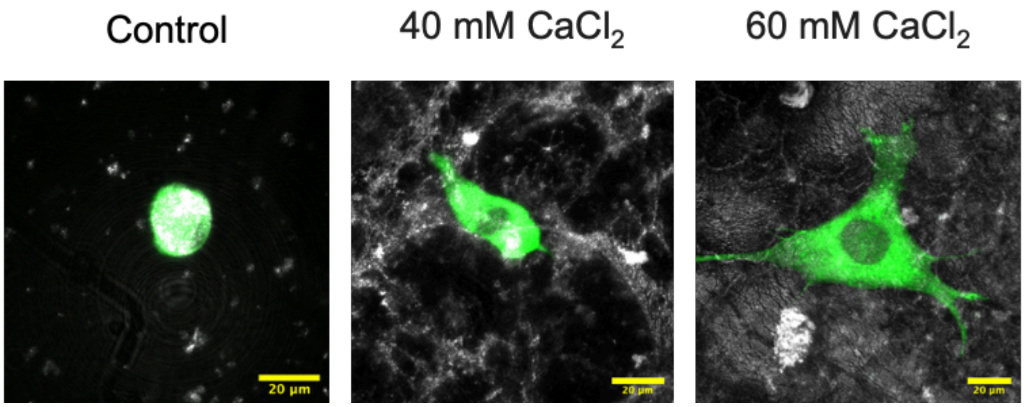

Figure S1: Representative single cells confocal images (maximum projection over a 20 µm depth) of NIH/3T3-dtomato cells embedded in ADA-GEL blends after three days in culture. Images show the superposition of the cell fluorescence signal (green) and the reflection (grey).

Figure S2

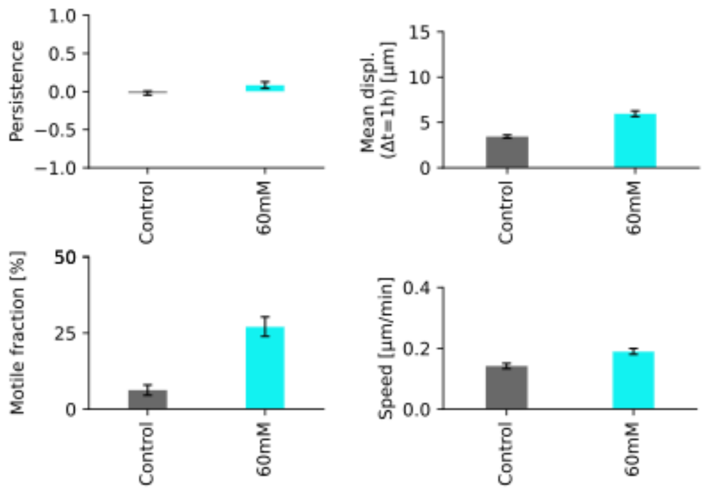

Figure S2: 24-hour migration (mean and standard error) of NIH/3T3 cells recorded starting immediately after seeding in terms of persistence, mean displacement, motile fraction, and speed, for the ADA-GEL control and ADA-GEL precrosslinked with 60 mM $CaCl_2$ (high).

Figure S3

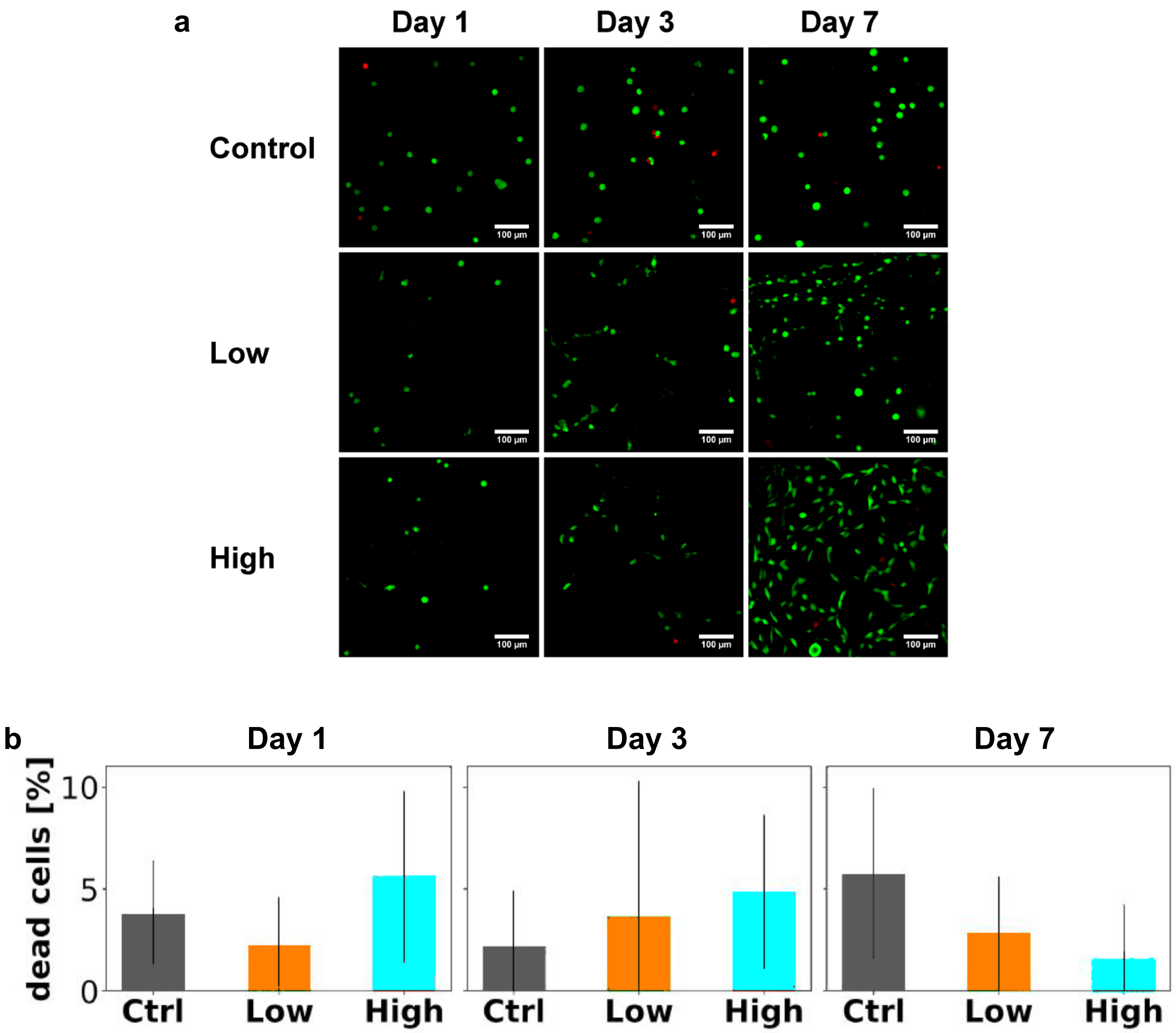

Figure S3: a, Maximum intensity projections of the live/dead assay of no pre-crosslinked (control), 40mM low pre-crosslinked (low) and 80 mM high pre-crosslinked (high) ADA-GEL. The images show fluorescent NIH3T3 cells stained with Calcein-AM (green) as live cells and EthD-1 (red) stained nuclei of dead cells. Each z-stack contains 60 images with a step size of 5µm and was taken at 10x magnification with a 20x objective and 0.5x coupler; **b**, The plots show the percentage of dead cells. Each bar shows the mean and standard deviation across all samples (n=10-20) of each condition. Conditions are shown in grey (control), green (low pre-crosslinking) and red (high pre-crosslinking).

Figure S4

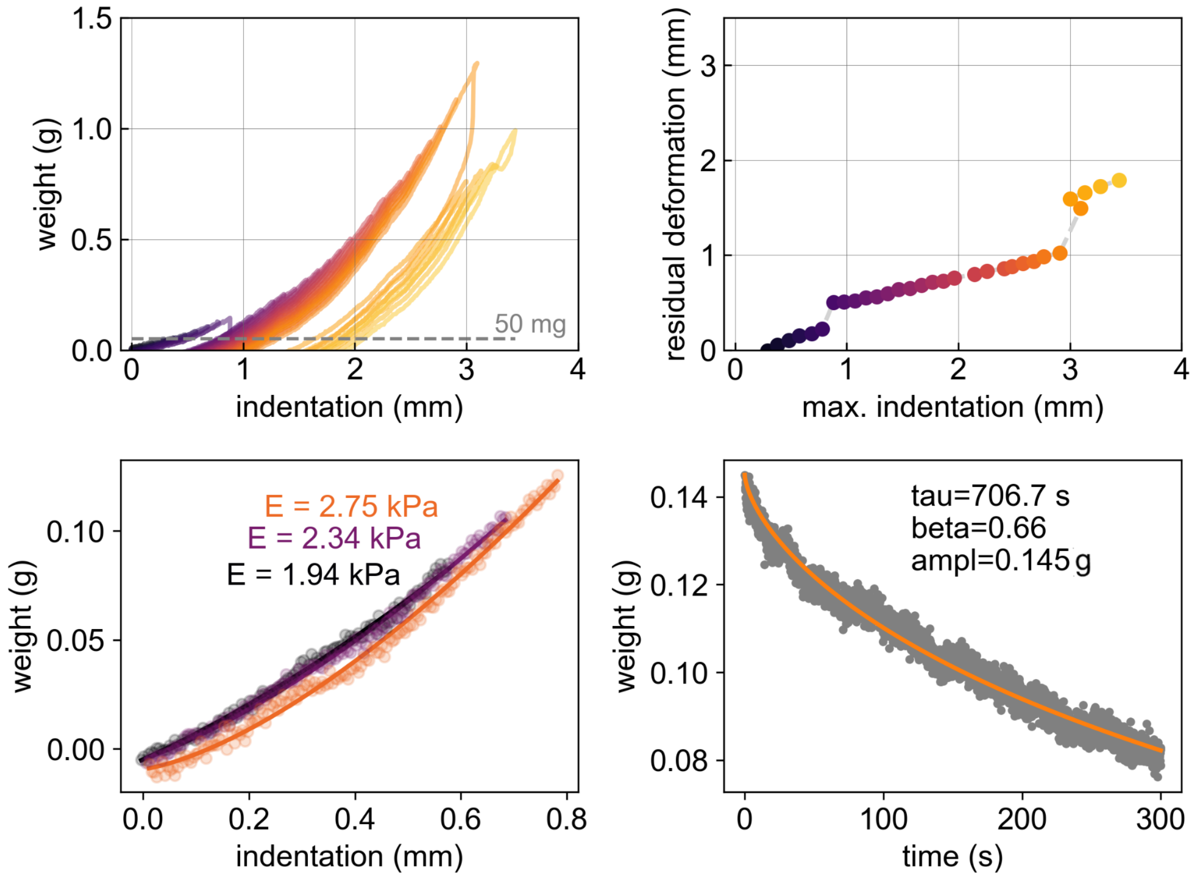

Figure S4: Representative profiles for the ADA-GEL pre-crosslinked with 20 mM $CaCl_2$, showing the weight vs. indentation curves as the indenter was inserted and withdrawn according to the indentation history shown in Fig. 6a (top left); the residual deformation at a threshold force (corresponding to a minimum 50 mg weight threshold) versus the maximum indentation for the specific cycles are coloured as in the previous weight vs. indentation curves (top right); the Young's modulus calculated by Hertz for cycles 5, 6 and 7 (bottom left), and the viscoelastic fit using a stretched exponential law (bottom right).

Figure S5

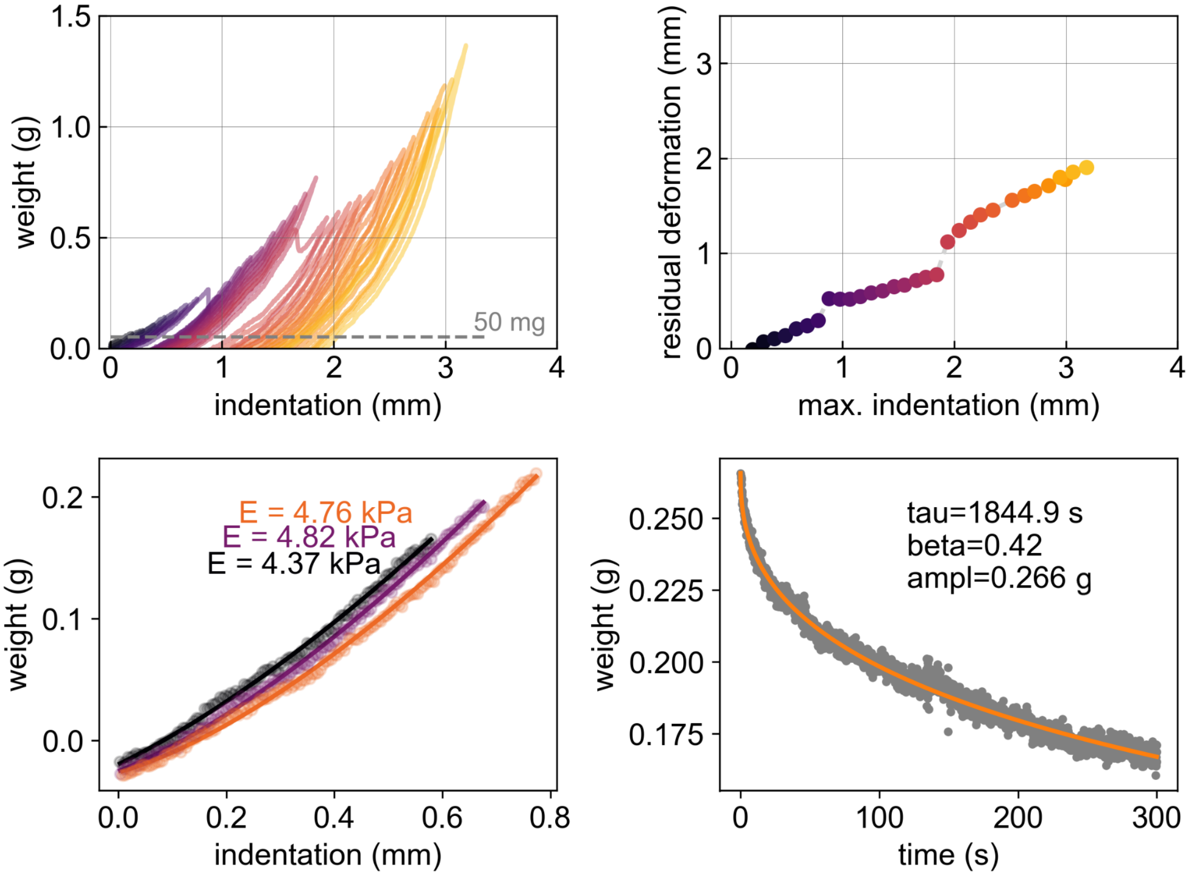

Figure S5: Representative profiles for the ADA-GEL pre-crosslinked with 40 mM $CaCl_2$, showing the weight vs. indentation curves as the indenter was inserted and withdrawn according to the indentation history shown in Fig. 6a (top left); the residual deformation at a threshold force (corresponding to a minimum 50 mg weight threshold) versus the maximum indentation for the specific cycles are coloured as in the previous weight vs. indentation curves (top right); the Young's modulus calculated by Hertz for cycles 5, 6 and 7 (bottom left), and the viscoelastic fit using a stretched exponential law (bottom right).

Figure S6

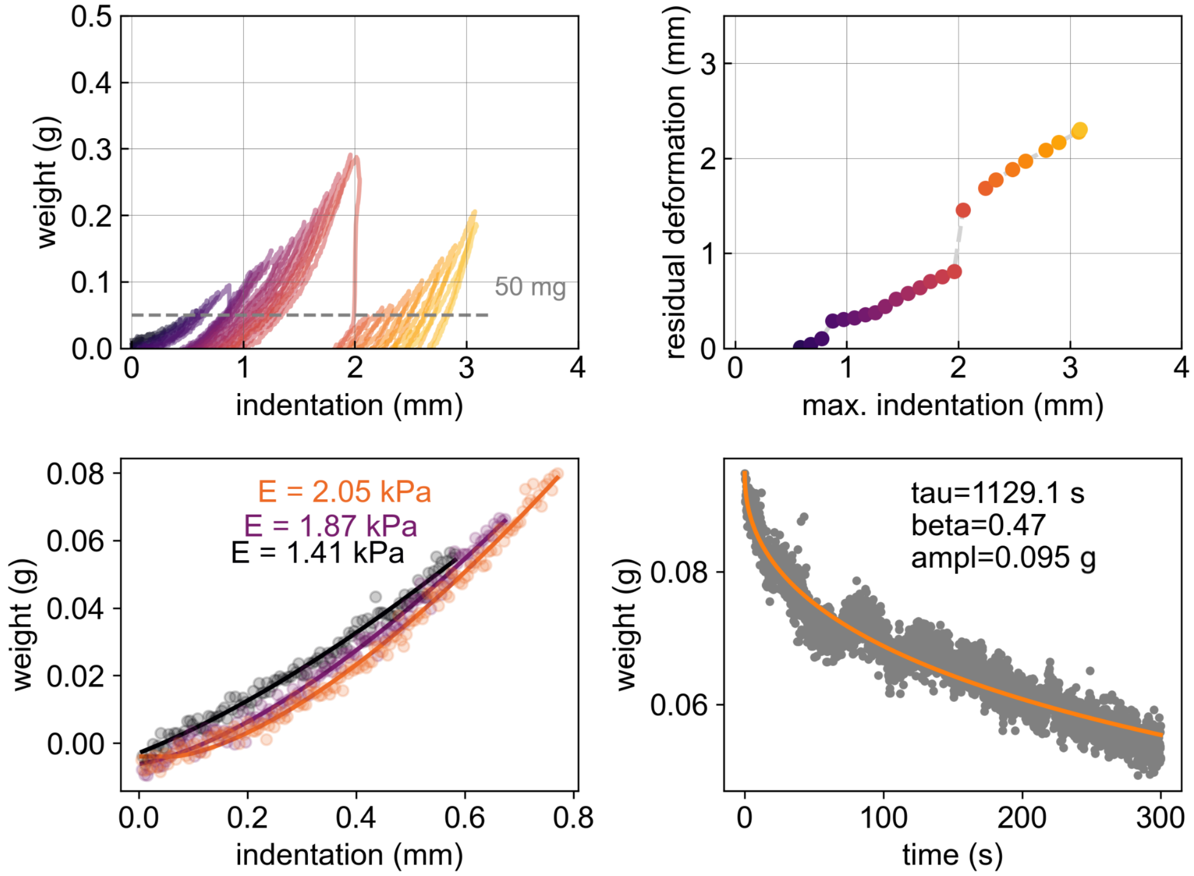

Figure S6: Representative profiles for the ADA-GEL pre-crosslinked with 60 mM $CaCl_2$, showing the weight vs. indentation curves as the indenter was inserted and withdrawn according to the indentation history shown in Fig. 6a (top left); the residual deformation at a threshold force (corresponding to a minimum 50 mg weight threshold) versus the maximum indentation for the specific cycles are coloured as in the previous weight vs. indentation curves (top right); the Young's modulus calculated by Hertz for cycles 5, 6 and 7 (bottom left), and the viscoelastic fit using a stretched exponential law (bottom right).

Figure S7

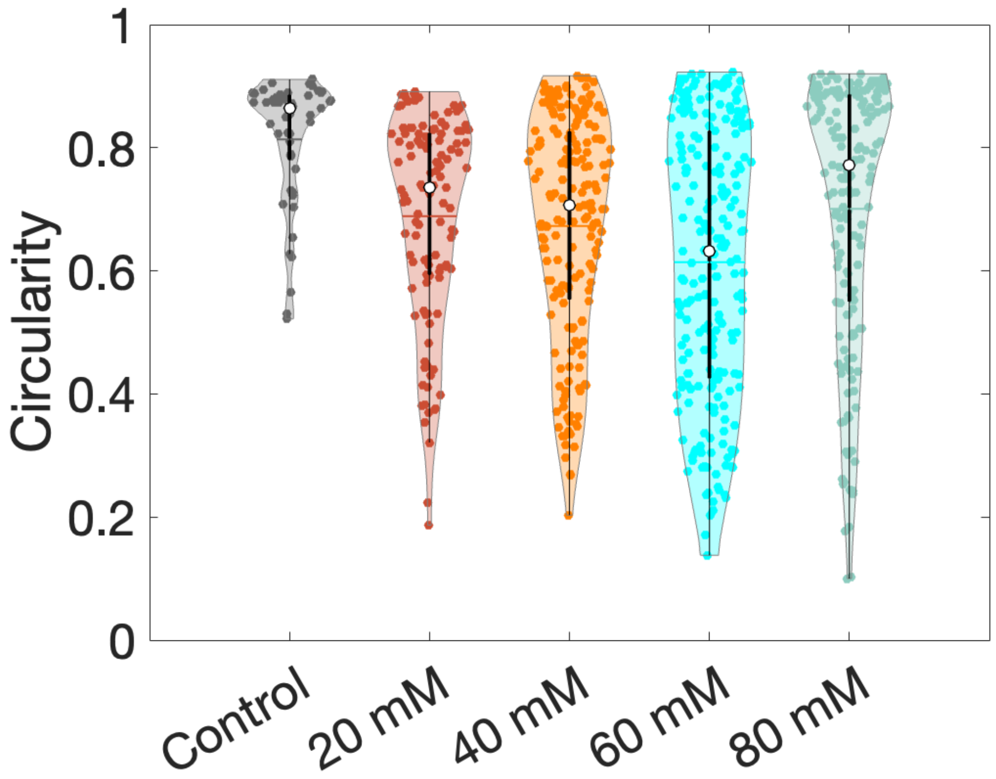

Figure S7: Violin plot of circularity of the NIH/3T3 cells for, control, and ADA-GEL pre-crosslinked with 20, 40, 60 and 80 mM $CaCl_2$, with mean (white dot) and bandwidth (black line) between the 10% and 40% data range, on days 1, 3 and 7.

Figure S8

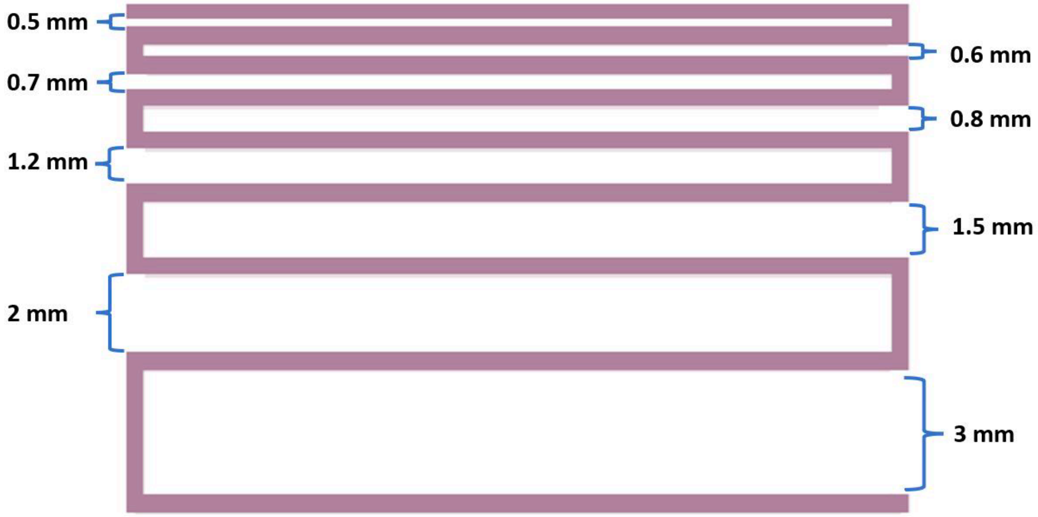

Figure S8: Schematic diagram of a printed three-dimensional structure for assessing the extrusion stability of hydrogels. The hydrogel material is printed onto a Petri dish.

Figure S9

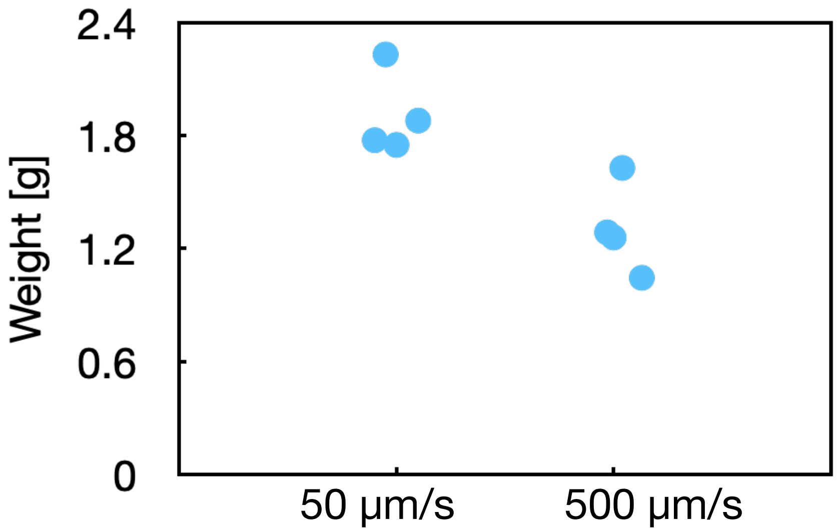

Figure S9: Puncture force (expressed as weight) of non-precrosslinked ADA-GEL hydrogel punctured with a 300 µm diameter indentation tip at two different displacement rates (n=4 replicates per condition). A paired t-test was performed with statistical significance of p=0.0052.

Figure S10

a

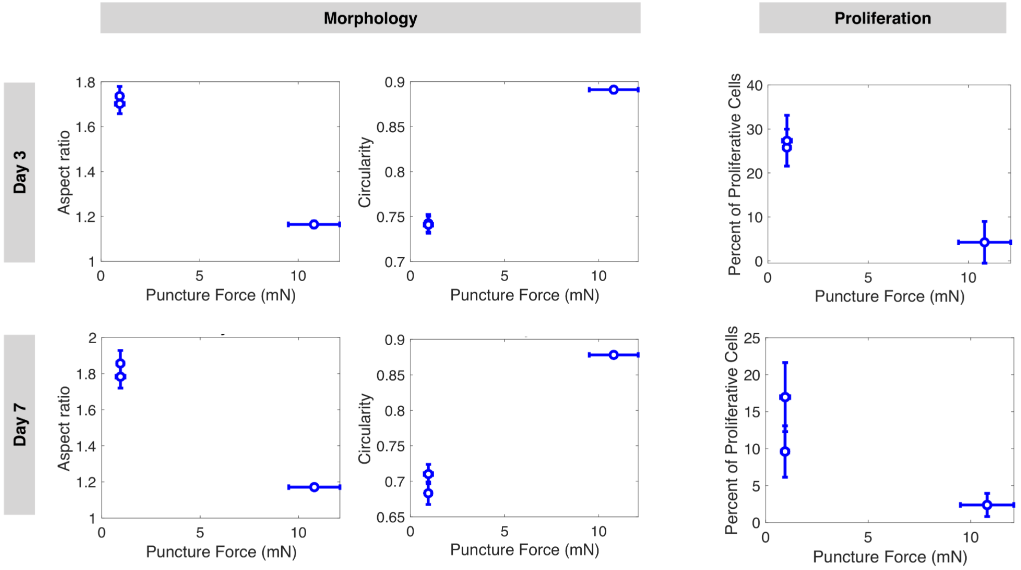

b

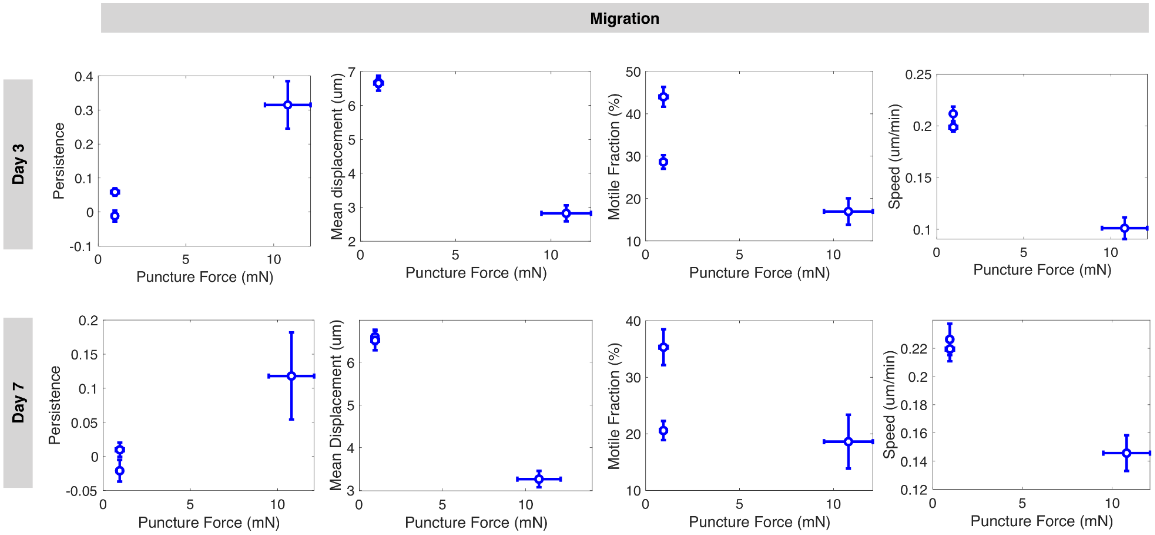

Figure S10: a, Correlation between puncture force with indentation diameter 300 μm (mean ± se) and biological results in terms of morphology (left) and proliferation (right) data (mean ± se) on day 3 (above) and day 7 (below), **b,** Correlation between puncture force with indentation diameter 300 μm (mean ± se) and biological results in terms of migration data (mean ± se) on day 3 (above) and day 7 (below).

Supplemental Video 1

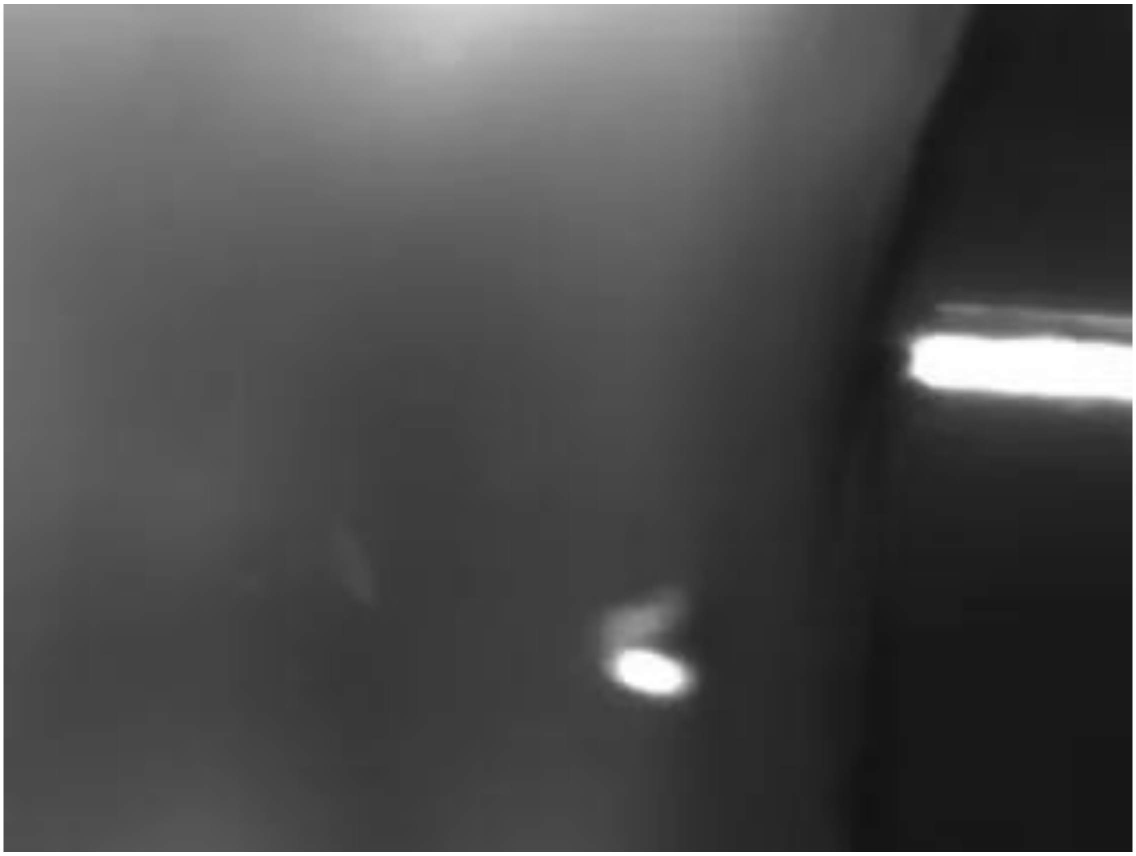

Supplemental Video 1: Puncture force experiment for the ADA-GEL control (non pre-crosslinked) condition with indentation diameter 300 µm. Video is taken in bright-field mode at 10 Hz with a CMOS camera (acA720-520um, Basler, Germany).

Supplemental Video 2

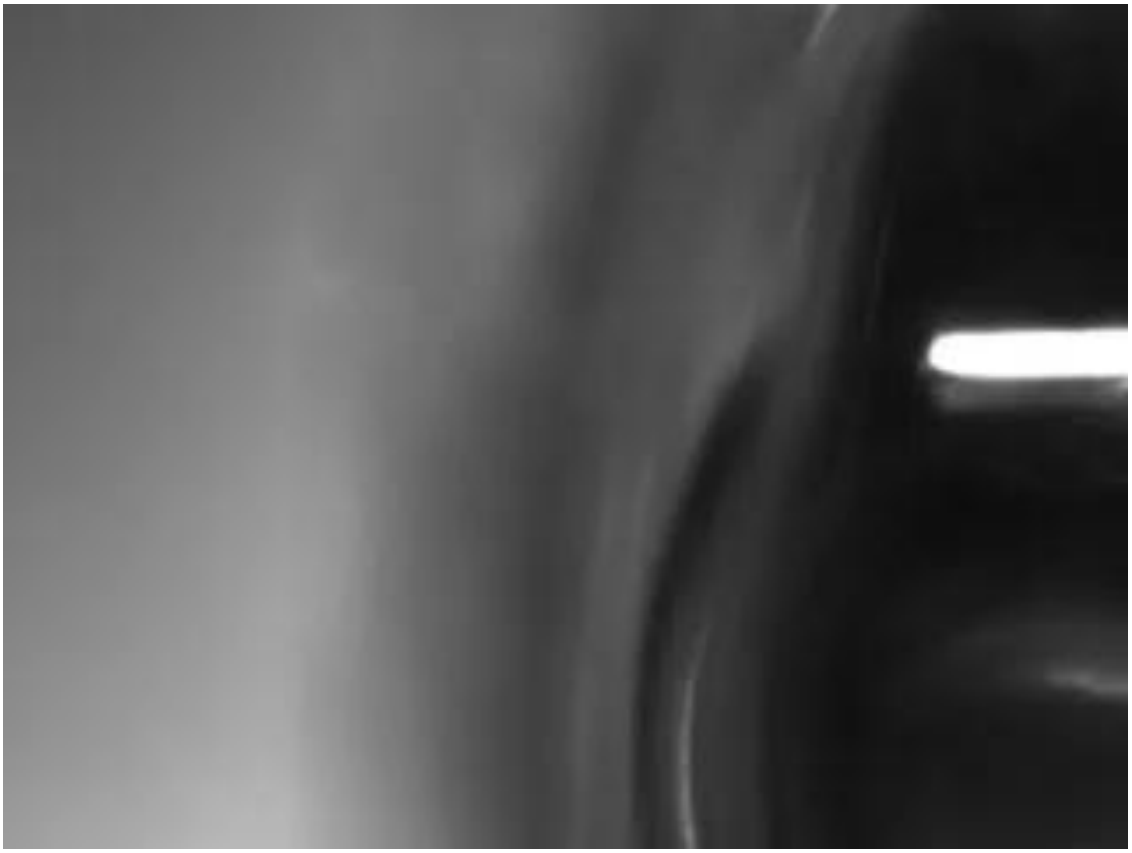

Supplemental Video 2: Puncture force experiment for the ADA-GEL 40 mM pre-crosslinked condition with indentation diameter 300 µm. Video is taken in bright-field mode at 10 Hz with a CMOS camera (acA720-520um, Basler, Germany).

Supplemental Video 3

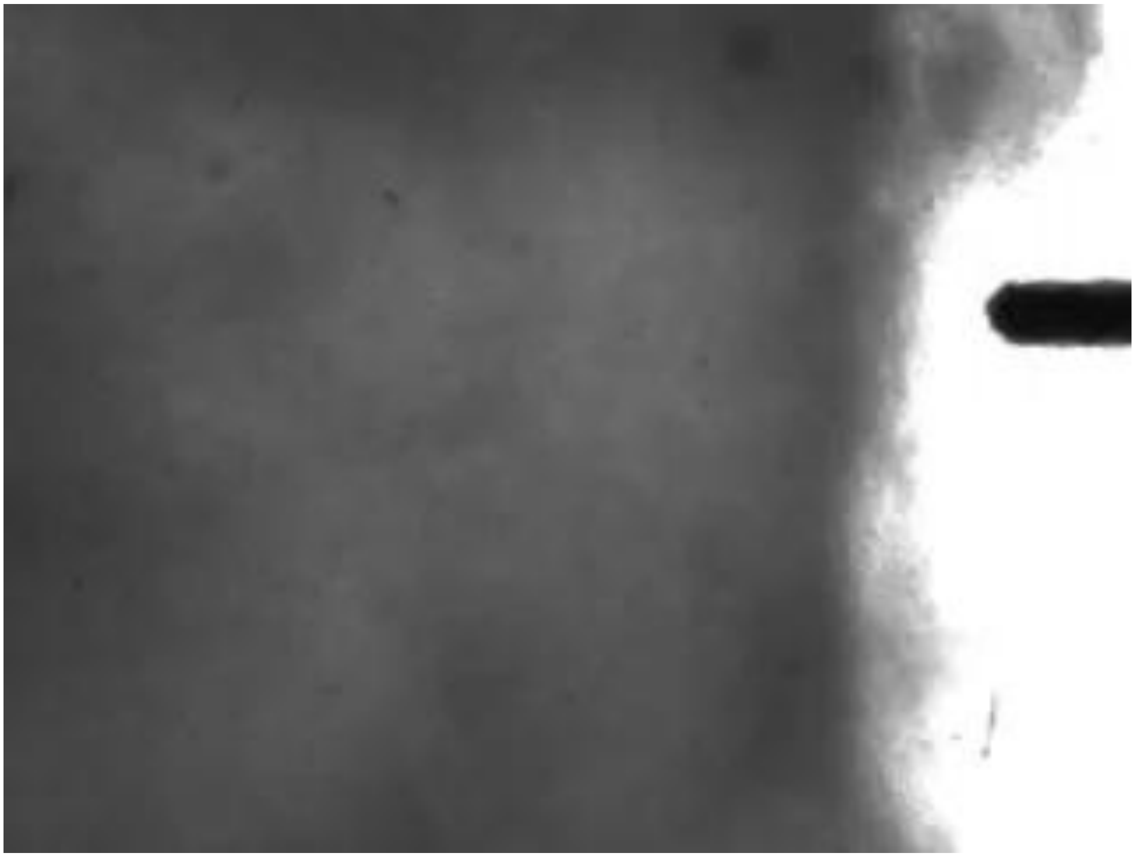

Supplemental Video 3: Puncture force experiment for the ADA-GEL 60 mM pre-crosslinked condition with indentation diameter 300 µm. Video is taken in bright-field mode at 10 Hz with a CMOS camera (acA720-520um, Basler, Germany).
