# Supplementary figures and images for: Plasticity of 3D Hydrogels Predicts Cell Biological Behavior
Source: Biomacromolecules. 2024 Nov 8;25(12):7608–18. doi: 10.1021/acs.biomac.4c00765 (PMC11632650; doi:10.1021/acs.biomac.4c00765)

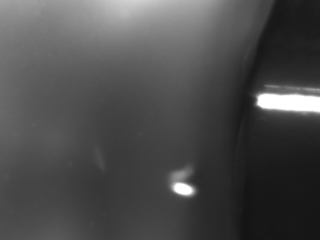

Supplement: Supplementary file 1 — bm4c00765_si_001.zip [file bm4c00765_si_001.zip › suppl-files/Control_ADA-GEL.gif]

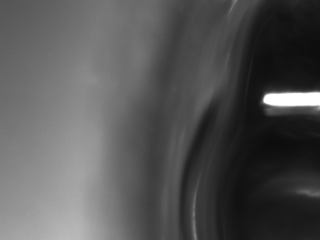

Supplement: Supplementary file 1 — bm4c00765_si_001.zip [file bm4c00765_si_001.zip › suppl-files/Precrosslinked_40mM_ADAGEL.gif]

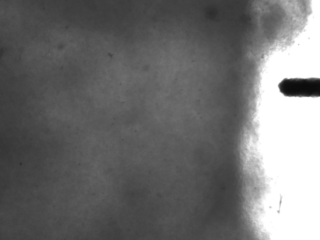

Supplement: Supplementary file 1 — bm4c00765_si_001.zip [file bm4c00765_si_001.zip › suppl-files/Precrosslinked_60mM_ADA-GEL.gif]
